# Rationally designed probiotics prevent shrimp white feces syndrome via the probiotics–gut microbiome–immunity axis

**DOI:** 10.1038/s41522-024-00509-5

**Published:** 2024-04-11

**Authors:** Haonan Sha, Jiaqi Lu, Jiong Chen, Jinbo Xiong

**Affiliations:** 1 State Key Laboratory for Managing Biotic and Chemical Threats to the Quality and Safety of Agro-products, Insititute of Plant Virology, Ningbo University, Ningbo, 315211 China; 2 School of Marine Sciences, Ningbo University, Ningbo, 315211 China

**Keywords:** Applied microbiology, Microbiome

## Abstract

Increasing evidence infers that some complex diseases are attributed to co-infection with multiple pathogens, such as shrimp white feces syndrome (WFS); however, there is a lack of experimental evidence to validate such causal link. This deficiency further impedes rational design of probiotics to elicit desired benefits to shrimp WFS resistance. Herein, we validated the causal roles of *Vibrio fluvialis*, *V. coralliilyticus* and *V. tubiashii* (in a ratio of 7:2:1) in shrimp WFS etiology, which fully satisfied Koch’s postulates. Correspondingly, we precisely designed four antagonistic strains: *Ruegeria lacuscaerulensis*, *Nioella nitratireducens*, *Bacillus subtilis* and *Streptomyces euryhalinus* in a ratio of 4:3:2:1, which efficiently guarded against WFS. Dietary supplementation of the probiotics stimulated beneficial gut populations, streptomycin, short chain fatty acids, taurine metabolism potentials, network stability, tight junction, and host selection, while reducing turnover rate and average variation degree of gut microbiota, thereby facilitating ecological and mechanical barriers against pathogens. Additionally, shrimp immune pathways, such as Fcγ R-mediated phagocytosis, Toll-like receptor and RIG-I-like receptor signaling pathways conferring immune barrier, were activated by probiotics supplementation. Collectively, we establish an updated framework for precisely validating co-infection with multiple pathogens and rationally designing antagonistic probiotics. Furthermore, our findings uncover the underlying beneficial mechanisms of designed probiotics from the probiotics–gut microbiome–host immunity axis.

## Introduction

Shrimp (*Litopenaeus vannamei*) holds a preeminent position in aquaculture, boasting the highest levels of scale, production, and economic value^1^. The intensive farming model exerts stresses on shrimp populations, leading to increased frequency and severity of diverse diseases. Among these diseases, white feces syndrome (WFS) is a multifactorial disease whose causal pathogens are currently uncertain, thus is named from the typical symptoms^2^. Accumulating evidence has revealed distinct gut bacterial communities between healthy and WFS shrimp^3–6^. Furthermore, the transplantation of the gut microbiota from WFS donors to healthy shrimp can induce an outbreak of WFS in the recipients^7^. These findings depict that dysbiosis in the gut microbiota is implicated in WFS etiology. Employing ecological approaches, our recent work infers three candidate pathogens of WFS: *Vibrio fluvialis*, *V. coralliilyticus* and *V. tubiashii*, which accurately distinguish WFS shrimp from healthy individuals^4^. Yet, experimental evidence that substantiates the causal link between the three pathogens and WFS occurrence is lacking.

Currently, efficient treatment targeting WFS is unavailable, while prophylactic antibiotics are proved to be ineffective and unsustainable. Thus, probiotics have arisen as a promising alternative for preventing and treating diseases. However, the beneficial effect of commercial probiotics is unstable or ineffectual in field^8^. One possible explanation is that external probiotics insufficiently colonize into shrimp gut^8^. Ecologically, application of strains isolated from the targeting host could be more efficient and effective than other sourced strains, given their adaptation to the natural defense systems of their hosts^9^. By this logic, probiotics sourced from shrimp gut have a better chance to successfully colonize into the gut, thereby improving shrimp fitness. Currently, the probiotics in aquaculture are primarily screened by blind isolation and subsequent antagonism assays, which might inadequately capture putative probiotics antagonizing pathogens via indirect exploitation mechanisms, e.g., competition of limited nutrients and niches^10^. Additionally, the combination of multiple probiotics is irrational, namely, the members of a probiotic cocktail are artificially selected from known probiotics with equal ratio.

Gut commensals regulate the colonization resistance against invaders, of which several keystone species exert disproportional roles in a community. Specifically, keystone species are featured by intensively and strongly biotic interactions with other community members, rather than their sheer abundance, thereby representing a vulnerable point in a community^11,12^. In accordance, the loss or removal of keystone species causes network fragmentation, leading to cascading aberrant alterations in community function and stability^13^. On this bias, supplementation of keystone species could contribute excellent potency in gut microbiota-based interventions^14,15^. Indeed, case studies have identified specific keystone strains to efficiently inhibit one pathogen, such as *Clostridium scindens*—*C*. *difficile*^16^, and *Bacillus subtilis*—*Staphylococcus aureus*^17^. By this logic, intervention targeting keystone species to antagonize multiple pathogens could precisely manipulate the gut microbiome, which could be a potent and precise tactic for shrimp WFS.

During host–pathogens interaction, the invading pathogens skew host gut microbiota and immunity in their favor. For example, the degrees of dysbiosis in the gut microbiota are positively associated with increasing WFS severity^5^, while a resilient gut microbiota alleviates shrimp mortality during *V*. *harveyi* infection^18^. To eliminate pathogens, WFS suffered shrimp activate innate immune pathways, such as NOD-like receptors (NLRs) signaling pathway and downstream NF-κB cascades. However, pathogens inhibit the expression of pathogens-associated molecular patterns to evade immune clearance, resulting in the development of shrimp WFS^19^. On the contrary, probiotics could block pathogens’ signaling system^20^, stimulate gut symbionts and host immunity^21^. So, the multidirectional interactions among multiple pathogens, the gut microbiota, and shrimp immune responses, known as gut microbiota-immunity axis, are implicated in WFS etiology. However, the exact interplay among probiotics-gut microbiome and crustaceans immunity is yet to be established^21^, thereby limiting mechanistic understanding of probiotics-based therapies.

Here, we validated the causality between shrimp WFS and co-infection with *Vibrio fluvialis*, *V. coralliilyticus* and *V. tubiashii* that were ecologically inferred in our prior work^4^. Going forward, we designed probiotics to antagonize the three WFS-causing pathogens using a causal interaction network^15^. To evaluate whether our designed probiotics efficiently potentiated shrimp WFS resistance, shrimp were fed with probiotics-supplemented diet for 14 days, and then were bath infected with the pathogenic cocktail. We sought to design, interrogate and validate antagonistic probiotics mediating WFS resistance. This is a valuable attempt to causally validate co-infection with multiple pathogens and precisely design correspondingly antagonistic probiotics. Furthermore, detailed ecological and molecular mechanisms that underlying the importance of probiotics were proposed from the probiotics-gut microbiome and host immunity axis.

## Results

### Validation of WFS causing pathogens

**Fig. 1:**
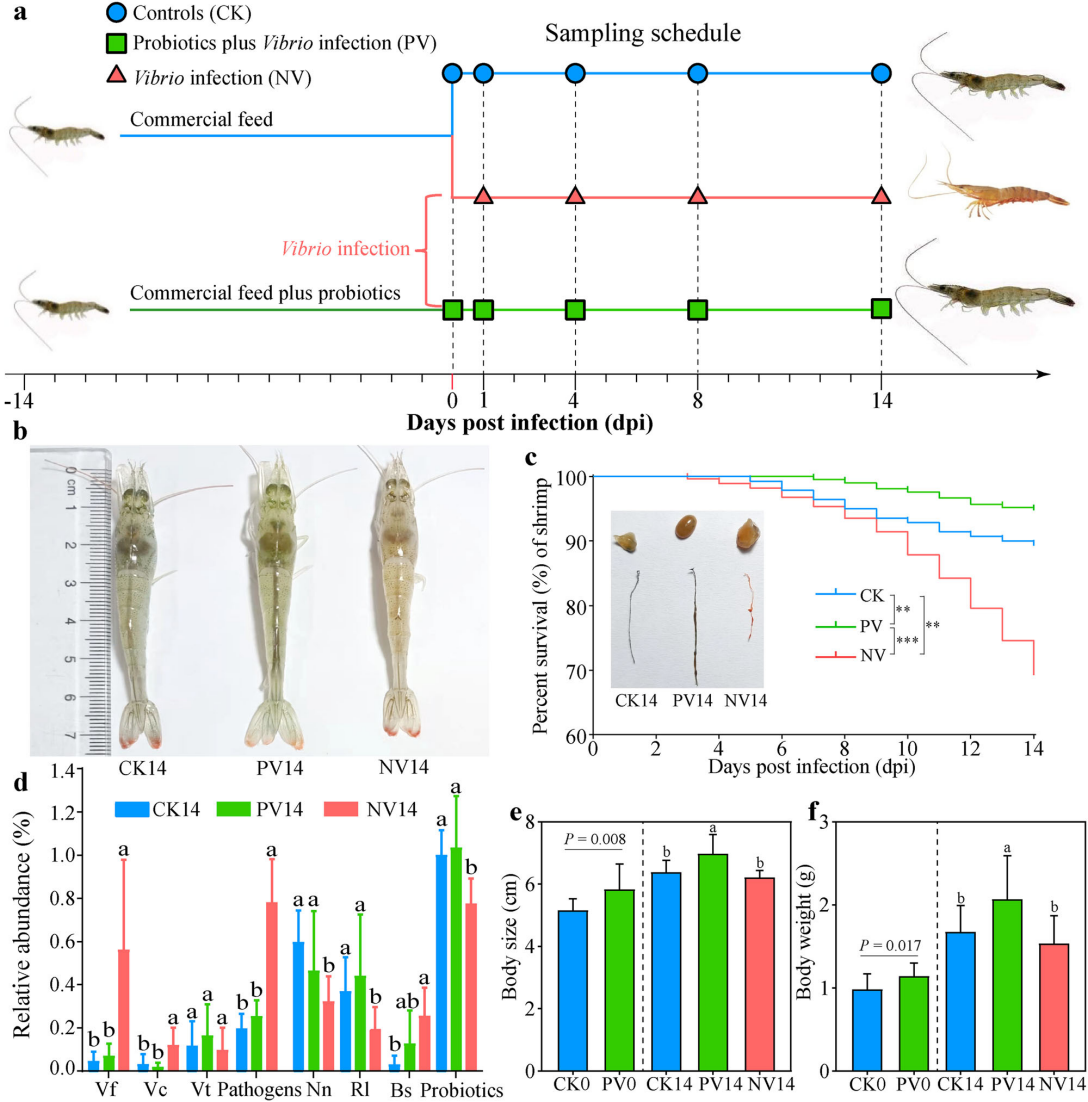
Dietary supplementation of designed antagonistic probiotics efficiently protects shrimp from white feces syndrome (WFS). **a** The experimental design. Shrimp were fed a basic diet supplemented with/without antagonistic probiotics (1 × 10^7^ CFU/g diet) for 14 days, and then were bath infected with *Vibrio fluvialis* (Vf), *V. coralliilyticus* (Vc) and *V. tubiashii* (Vt) (with a ratio of 7:2:1) at a density of 1 × 10^8^ CFU/mL. **b** Shrimp phenotypes among treatments on 14 days post infection (dpi). **c** Kaplan–Meier graph of shrimp mortality along dpi. **and *** indicate significant difference between different treatments at *P* < 0.01 and *P* < 0.001 levels by using log-rank comparison. **d** Comparing relative abundances (mean ± standard deviation) of the three pathogens, summed pathogens, the detected antagonistic strains, *Ruegeria lacuscaerulensis* (Rl)*, Nioella nitratireducens* (Nn), *Bacillus subtilis* (Bs), and summed probiotics in gut among treatments on 14 dpi. **e** Shrimp body length (mean ± standard deviation), **f** Shrimp weight (mean ± standard deviation) on 0 dpi (before infection) using unpaired *t* test, and on 14 dpi using one-way analysis of variance (ANOVA). Different lowercase letters indicate significant differences among treatments.

The co-infection with *V. fluvialis* (Vf), *V. coralliilyticus* (Vc) and *V. tubiashii* (Vt) caused typical WFS symptoms, such as sluggishness, reduced feeding, atrophic intestine and massive mortality (Fig. 1b, c). However, neither the infection of single *Vibrio* strain nor paired *Vibrio* strains of Vf, Vc and Vt could reproduce these syndromes (Supplementary Fig. 1). Additionally, the three *Vibrio* pathogens were detected in gut, which significantly enriched in NV (*Vibrio* infection without probiotics supplementation) shrimp compared with control shrimp (CK) (Fig. 1d). Collectively, the three *Vibrio* pathogens were isolated from WFS-infected shrimp, synergistically caused WFS symptoms, and enriched in re-infected individuals, which fully satisfied Koch’s postulates. Thus, Vf, Vc and Vt were the causing pathogens of WFS. However, we can’t completely refuse other pathogens are implicated in shrimp WFS. Further work is required to resolve the etiology of WFS.

### Designing antagonistic probiotics against WFS

**Fig. 2:**
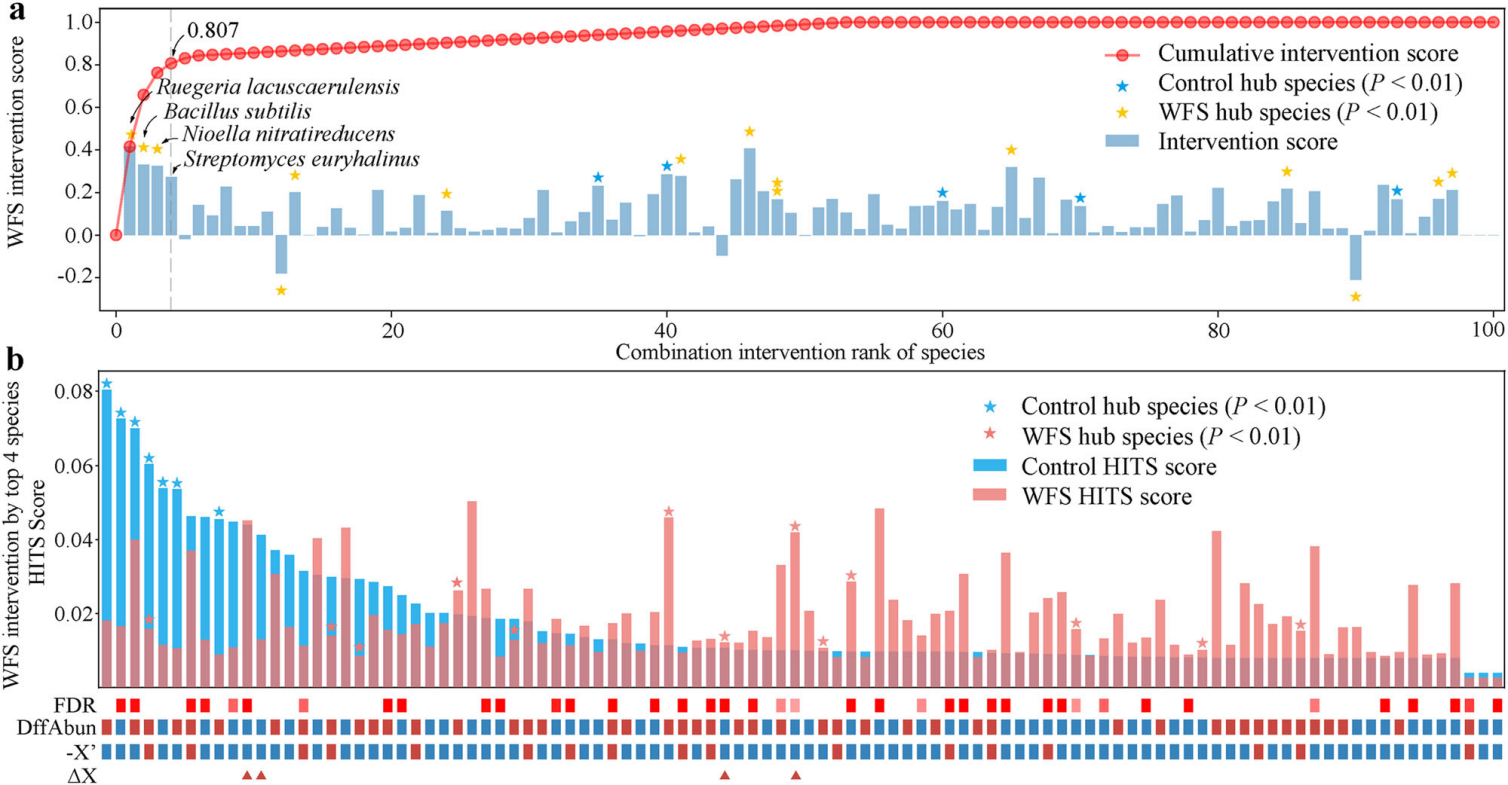
Designing antagonistic probiotics to prevent shrimp WFS using dynamic intervention simulation (DIS). **a** Intervention scores (ISs) of the top 100 gut symbionts. The IS of each taxon is shown in the bar plot and the stars with the blue and red color indicate the significance of the Hyperlink‐Induced Topic Search (HITS) score in the gut microbiota of healthy and WFS cohorts, respectively. Negative IS indicates a change away from the microbiome of healthy shrimp. The red curve indicates the combination intervention scores (CISs) of the microbes sequentially selected by DIS, which represents the intervention effect after the simultaneous intervention of multiple species. The top four keystone species, achieving a CIS > 0.8, are indicated. **b** Effect of microbial intervention on the WFS gut microbiota according to DIS with the four antagonistic species. HITS scores of species in the gut microbiota of healthy shrimp are sorted in the bar plot, and the hub species are marked with stars. DiffAbun: abundance change from healthy to WFS shrimp (red: increase, blue: decrease), with the false discovery rate (FDR) are indicated above (red: FDR < 0.01, light red: FDR < 0.05). −*X*′: negative representation of instant bacterial abundance changes upon the intervention (red: −*X*′ > 0, blue: −*X*′ < 0). The top four antagonistic taxa for intervention are marked by triangles in ∆*X*.

Given that keystone species are excellent targets for gut microbiota-based interventions^14,15^, we determined the optimal combination of keystone species with the greatest combination intervention score (CIS) using Iterative Feature Elimination (IFE). IFE, a feature selection strategy based on the greedy algorithm^22^, was used to search for the optimal combination of microbial species for the intervention. CIS represents the intervention effect after the simultaneous intervention of the optimal combination of microbial species, thus a higher CIS value indicates a better intervention effect^15^. The top four keystone taxa from the gut microbiota of WFS shrimp contributed a CIS of 0.807 (Fig. 2a and Supplementary Table 1). Targeting the four keystone taxa in the WFS shrimp induced restorations in members that were the hub taxa in the gut microbiota of healthy shrimp (Fig. 2b). Hence, the four keystone taxa maximally regained toward a healthy gut microbiota, which were selected as antagonistic probiotics for preventing WFS.

Mortality in NV shrimp occurred on 3 days post infection (dpi) and afterward. By contrast, owing to the probiotics supplementation for 14 days, the fist mortality in PV (probiotics plus *Vibrio* infection) shrimp was observed on 7 dpi, which delayed by 4 days compared with NV shrimp (Fig. 1a, c). Notably, the cumulative mortality rate in PV shrimp was 5.2% on 14 dpi, which was significantly lower than that in the CK (10.7%) or NV (30.4%) shrimp (Fig. 1c). The antagonistic probiotics were isolated from healthy shrimp, thus were detected in CK group with relatively high abundances (Fig. 1d). As anticipated, significant and negative associations were detected between the summed relative abundance of the antagonistic probiotics and that of the three *Vibrio* pathogens (Supplementary Figure 2). In addition, probiotics supplementation increased shrimp pepsin and lipase activities on 0 dpi (Supplementary Fig. 3a, b), and accordingly improved body weight and body length compared with CK shrimp (Fig. 1e, f). After 14 days of infection, PV shrimp exhibited significantly higher immune and digestive activities than NV shrimp, with the exception of acid phosphatase (Supplementary Fig. 3). Collectively, our designed probiotics efficiently protect shrimp from WFS, improve shrimp survival, immunity and production.

### Responses of bacterial community along days post infection

Intrigued by the distinct phenotypes (Fig. 1), we next sought to elucidate any compositional differences in the gut microbiota among treatments. After rarefication to 16,019 sequences per sample, there were 2195 amplicon sequence variants (ASVs) across the 120 enrolled samples (Supplementary Table 2). Both probiotics supplementation and pathogens infection significantly altered the shrimp gut microbiota and bacterioplankton communities (Supplementary Fig. 4a, b). However, probiotics supplementation counteracted the effect of pathogens infection on the gut microbiota to a certain extent, as supported by gradual separations along axis 1 (Supplementary Fig. 4a). In accordance, striking differences in the abundances of dominant genera were observed between NV and CK shrimp, with moderate changes of these genera in PV shrimp on 14 dpi (Supplementary Fig. 5). Three out of the four antagonistic strains were detected in gut, whose relative abundances in NV shrimp were significantly lower than these in CK or PV cohort on 14 dpi (Fig. 1d). Although *Streptomyces euryhalinus* was undetectable on 14 dpi (Fig. 1d), the relative abundance of genus *Streptomyces* in PV gut bacterial community was significantly higher than that of NV (Supplementary Fig. 5a). In accordance, the effectiveness of probiotics extends beyond their viability, instead, the host could benefit from the metabolites and cellular components of probiotics^23^. Permutational multivariate analysis of variance (PERMANOVA) revealed that dpi, probiotics and infection explained 6.3%, 4.7% and 5.2% (*P* < 0.001 in each case) variance in the gut bacterial communities, respectively (Supplementary Table 3).

**Fig. 3:**
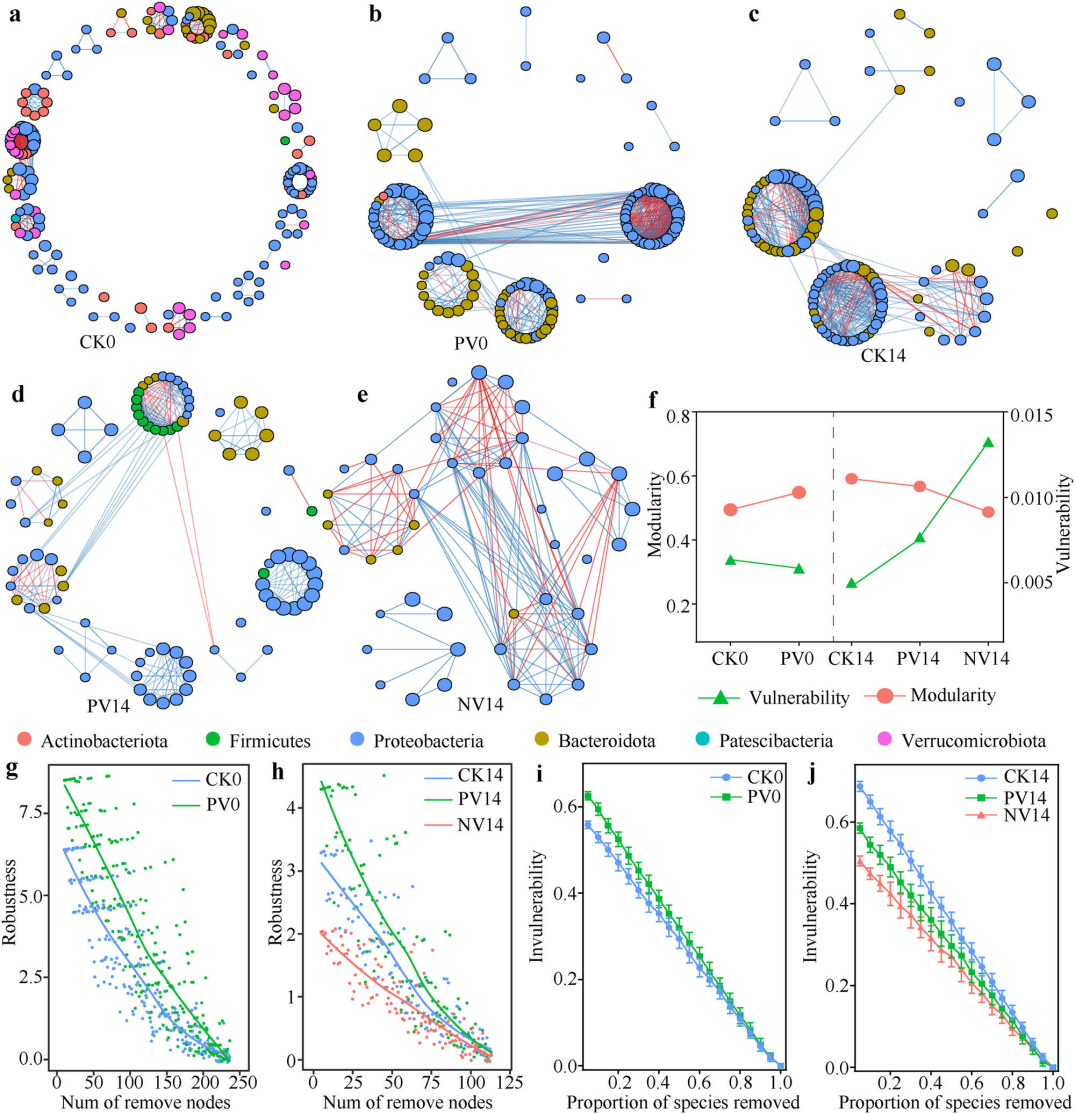
Antagonistic probiotics and pathogens infection effects on the biotic interactions among gut commensals. Co-occurrence networks for, **a** CK (control shrimp), **b** PV (probiotics plus *Vibrio* infection) shrimp on 0 dpi, **c** CK, **d** PV, **e** NV (*Vibrio* infection without probiotics supplementation) shrimp on 14 dpi, respectively. Each node (point) represents individual ASV, which is colored by its affiliated phylum. The size of a node represents its relative abundances in each bacterial community. Edges represent significant Spearman correlations (|*r*| > 0.8 and *P* < 0.05, Spearman’s rank correlation coefficient test), with red and blue lines indicate negative and positive correlations, respectively. Network stability between different treatments was compared by using, **f** modularity and vulnerability, **g** robustness on 0 dpi, **h** robustness on 14 dpi, **i** invulnerability on 0 dpi, **j** invulnerability on 14 dpi, respectively.

The temporal turnover rate of gut microbiota in CK shrimp (slope = −0.028) was significantly (*P* = 0.044, two-way analysis of variance) lower than that in NV shrimp (slope = −0.060) (Supplementary Fig. 4c), suggesting that pathogens infection accelerated the replacement of gut commensals over WFS progression. However, temporal turnover rate was attenuated by probiotics supplementation, with the lowest slope in PV shrimp (slope = −0.007) (Supplementary Fig. 4c). The stability of gut microbiota was evaluated using average variation degree (AVD). A lower AVD value indicates a higher community stability^24^. AVD values were comparable between PV and CK shrimp on 0 dpi (Supplementary Fig. 4d), indicating that probiotics colonization did not disrupt the stability of shrimp gut microbiota. In contrast, AVD in PV shrimp was significantly lower than that in NV cohort on 14 dpi (Supplementary Fig. 4d), thus antagonistic probiotics counteracted the instability of gut microbiota imposed by infection. Consistently, Bugbase inference revealed that pathogens infection increased the potentials of anaerobic, forms biofilms and putative pathogens in NV shrimp compared with CK shrimp (Fig. 3e−g). Of note, the potential of putative pathogens was the lowest in PV shrimp among the three groups (Fig. 3g).

### Ecological processes governing shrimp gut microbiota

An increased stochasticity governing host gut microbiota favors the invasion of external pathogens^25^, we therefore elevated whether probiotics supplementation potentiated the importance of determinism underlying the shrimp gut microbiota using a neutral community model (NCM)^26^. NCM assumes that bacterioplankton community is a species pool for the shrimp gut microbiota. The overall fit to NCM in CK shrimp (*R*^*2*^ = 0.777, Supplementary Fig. 6a) was lower than that in NV shrimp (*R*^*2*^ = 0.786, Supplementary Fig. 6c), thus pathogens infection enhanced stochasticity underlying the shrimp gut microbiota. However, probiotics supplementation mitigated infection effect, with a *R*^2^ value of 0.726 in PV shrimp (Supplementary Fig. 6b). Consistently, 93.5% of the total ASVs were neutrally distributed between NV shrimp gut and their rearing water, which was much higher than the proportions in PV (50.5%) and CK (50.1%) cohorts. Conversely, the proportion of overrepresented or underrepresented ASVs exhibited the opposite trend (Supplementary Fig. 6a−c).

We further detailed the ecological processes into the five ecological processes using the iCAMP model (Supplementary Fig. 6d−f). The relative importance of homogenizing selection governing the gut microbiota in CK shrimp was 33.9% (Supplementary Fig. 6d), which decreased to 24.4% in NV shrimp (Supplementary Fig. 6f). By contrast, the contribution of homogenizing selection sharply increased to 60.2% in PV shrimp (Supplementary Fig. 6e). Reciprocally, drift emerged as the predominant ecological process in NV shrimp (62.2%), whereas this process dropped to 17.8% in PV shrimp (Supplementary Fig. 6e, f).

### Effects of probiotics and pathogens infection on the networks of gut microbiota

**Fig. 4:**
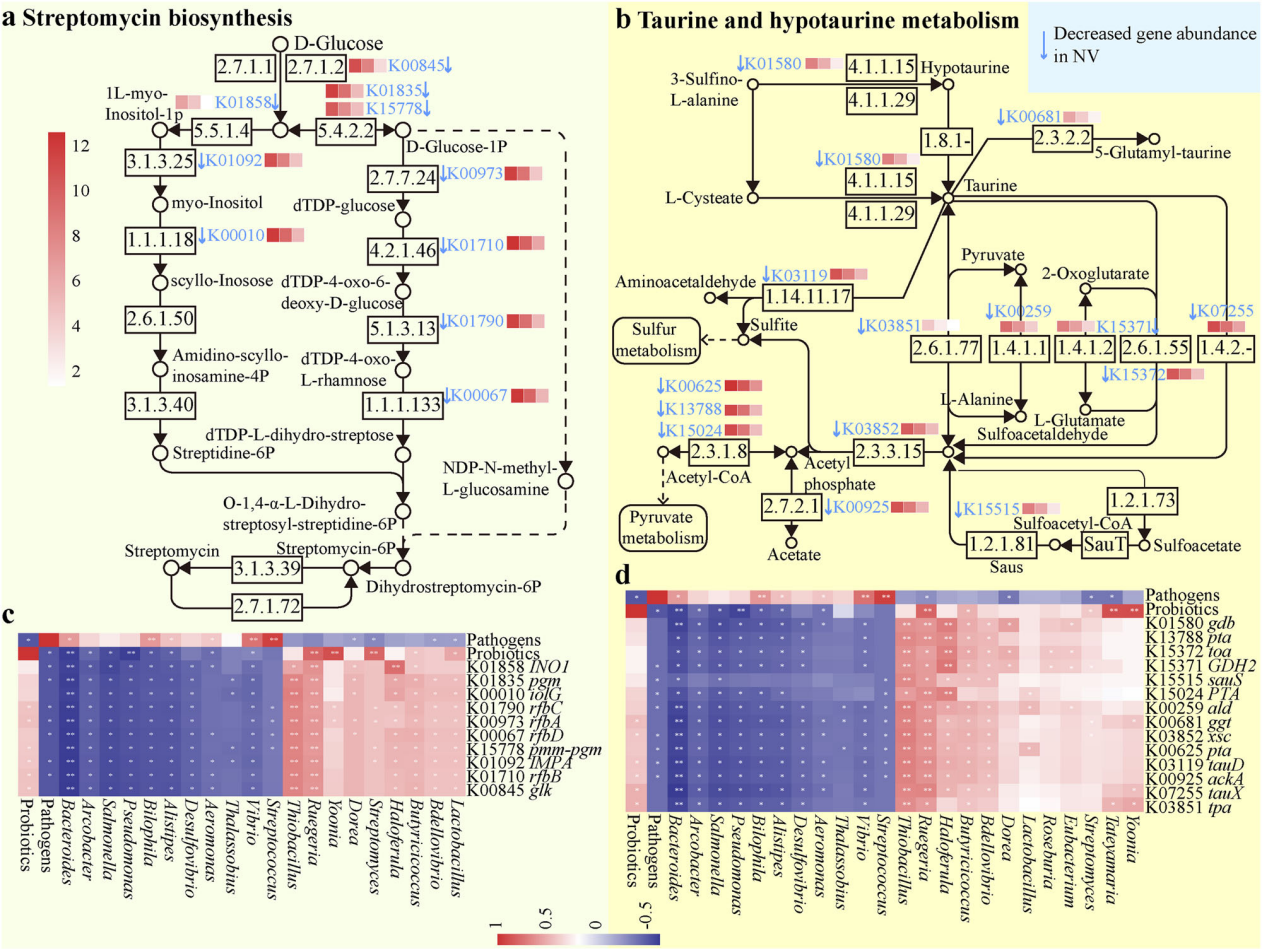
Mapped pathways involving in anti-infection in shrimp gut microbiome. **a** Streptomycin biosynthesis. **b** Taurine and hypotaurine metabolism. The downward arrows indicate the differentially functional pathways are depressed in NV shrimp. The gut bacterial genera, summed abundance of pathogens, and summed abundance of probiotics exhibit strong associations with the genes involving in, **c** streptomycin biosynthesis, **d** taurine and hypotaurine metabolism. Only the Spearman rank correlations with *P* < 0.05 are considered as strong associations and are marked with “*” in the heatmap.

To determine the effect of probiotics and infection on the biotic interactions among gut commensals, we constructed co-occurrence networks for each group on 0 dpi and 14 dpi, respectively (Fig. 4). PV shrimp exhibited higher network stability than CK shrimp on 0 dpi, as supported by lower vulnerability, higher modularity and robustness (Fig. 4). However, pathogens infection markedly reduced network robustness compared with CK shrimp on 14 dpi. This destabilized effect was reversed by probiotics supplementation, with the highest robustness in PV shrimp (Fig. 4h). After random removal of some nodes, the proportion of remaining species in the network of PV shrimp was consistently higher than that of CK shrimp (Fig. 4i), indicating network invulnerability was strengthened by probiotics supplementation. Again, antagonistic probiotics neutralized network invulnerability, modularity and robustness that were disrupted by pathogens infection (Fig. 4f, j).

### Effects of probiotics on functional potentials against infection

To further understand the beneficial effects of probiotics on shrimp–gut microbiome interaction against WFS, we next explored the differences in functional potentials among the three treatments. After quality control, 171.2 GB high-quality reads were generated for the 18 shrimp gut microbiomes on 14 dpi, of which 92.7% metagenomic reads passed the Q30 threshold (Supplementary Table 4). Compared with CK shrimp, the abundances of 163 pathways, including map00650 Butanoate metabolism, map00640 Propanoate metabolism and map00061 Fatty acid biosynthesis, significantly (*P* < 0.05, unpaired two-tail *t* test) decreased in NV shrimp on 14 dpi. By contrast, 57 pathways, such as map04620 Toll-like receptor (TLR), map04150 mTOR and map04630 JAK-STAT signaling pathways, exhibited the opposing trend (Supplementary Figure 7a). Similarly, 39 pathways conferring map00521 streptomycin biosynthesis, map00121 Secondary bile acid biosynthesis and map00650 Butanoate metabolism decreased significantly (*P* < 0.05, unpaired two-tail *t* test), whereas 6 pathways such as map00640 Propanoate metabolism and map04625 C-type lectin receptors (CLR) signaling pathway, were enriched in NV shrimp compared with PV shrimp on 14 dpi (Supplementary Fig. 7b). However, only three differential pathways were detected between CK and PV shrimp (Supplementary Fig. 7c), thus the functional structures of gut microbiome were comparable between the two cohorts. In other words, probiotics supplementation substantially alleviated the adverse effects imposed by pathogens infection.

There were intensive associations among pathogens, probiotics, and the gut microbiome (Supplementary Fig. 7d, e). *Vibrio* pathogens stimulated pathways facilitating pro-inflammatory responses, such as map04064 NF-κB, map04620 TLR, map04621 NOD-like receptor (NLR) and map04625 CLR signaling pathways. By contrast, probiotics positively affected diverse key metabolism pathways, including map00430 Taurine and hypotaurine, map00620 Pyruvate, map00640 Propanoate and map00650 Butanoate metabolisms (Supplementary Fig. 7d, e). Of note, the three *Vibrio* pathogens positively interacted with each other (Supplementary Fig. 7e), reinforcing their synergetic roles in shrimp WFS etiology.

Impressively, by aligning the altered genes to the KEGG database, we structured pathways involving in streptomycin biosynthesis (Fig. 3a), taurine and hypotaurine metabolism (Fig. 3b), propanoate metabolism (Supplementary Fig. 8), and butanoate metabolism (Supplementary Figure 9). The abundances of these implicated genes markedly decreased in NV shrimp compared with CK individuals. Still, the extent and severity of these reductions were less pronounced in PV than in NV shrimp. Of note, the abundances of the involved genes were positively associated with the beneficial genera of *Bdellovibrio*, *Butyricicoccus*, *Eubacterium*, *Clostridium* and *Streptomycete*, and the summed abundance of probiotics, whereas were negatively affected by detrimental *Vibrio*, *Pseudoalteromonas* and *Streptococcus* genera, and the summed abundance of the three *Vibrio* pathogens (Fig. 3, Supplementary Figs. 8 and 9).

### Significantly altered shrimp transcriptomes essential for WFS resistance

**Fig. 5:**
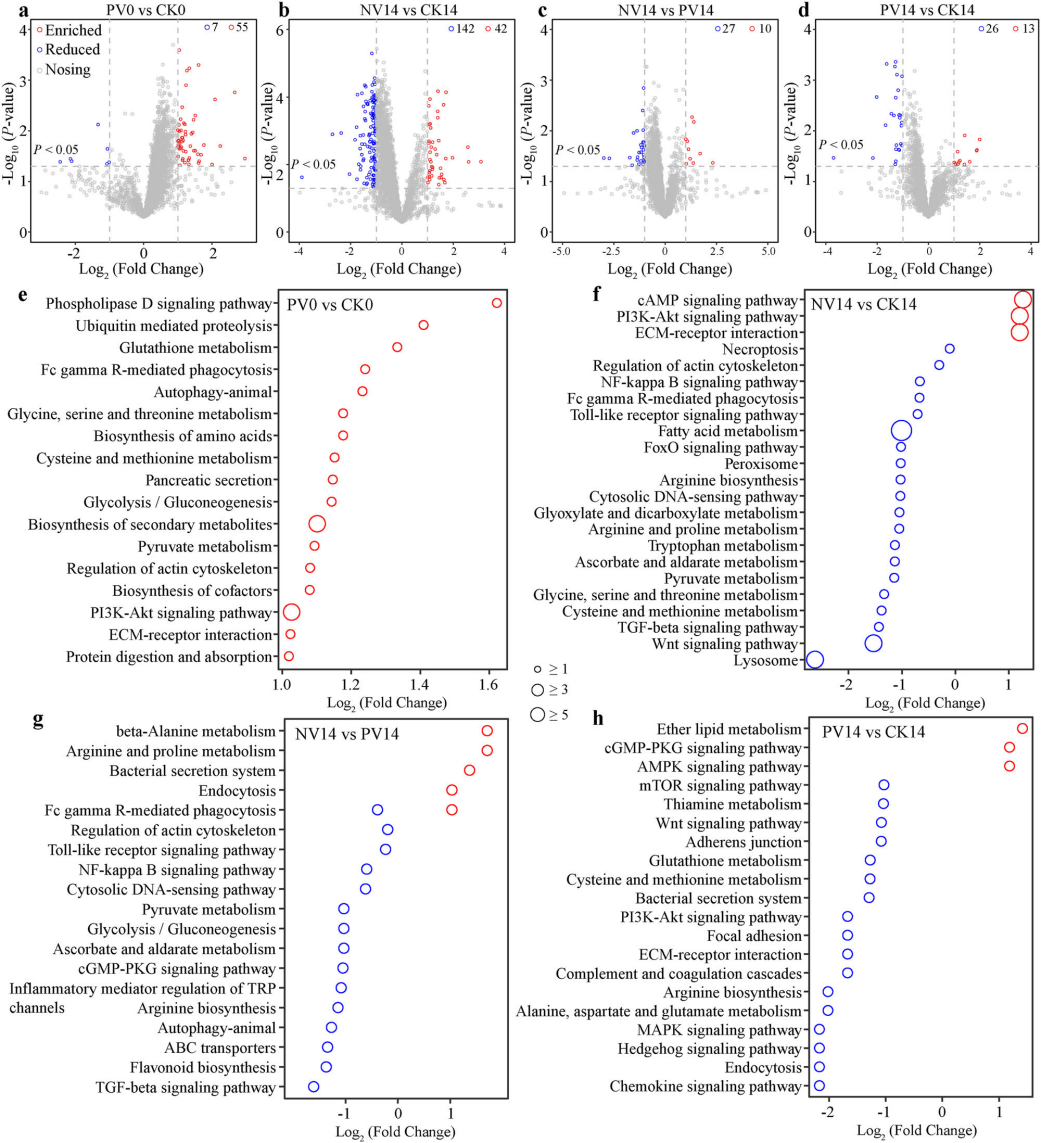
Antagonistic probiotics and pathogens infection effects on shrimp gut transcriptomes. Volcano plots depicting the distribution of differentially expressed genes (DEGs, **a**–**d**) and their corresponding pathways (**e**–**g**) between PV vs. CK (**a** or **e**) shrimp on 0 dpi, NV vs. CK (**b** or **f**), NV vs. PV (**c** or **g**), PV vs.CK (**d** or **h**) shrimp on 14 dpi using unpaired two-tail *t* test, respectively. Numbers indicate the up-regulated and down-regulated DEGs. Red and blue circles respectively indicate up-regulated or down-regulated pathways, while diameters are proportional to the detected number of differentially expressed genes in a given pathways.

Given the debate that the abundance of mRNA is unequal to activity, thus lipase, lysozyme or alkaline phosphatase activity was regressed against the mRNA level of corresponding encoding gene, respectively. As expected, positive and significant association was detected between the enzyme activity and expression of the matched coding gene (Supplementary Fig. 10). Hence, the altered expressive profiles in shrimp could be, at least partially, indicative of activities. Transcriptomes analysis revealed that 55 and 7 genes were up-expressed and down-expressed in PV shrimp compared with CK shrimp on 0 dpi, respectively (Fig. 5a). Probiotics supplementation significantly induced genes involved in biosynthesis of secondary metabolites, puruvate and glutathione metabolisms, FcγR-mediated phagocytosis, and regulation of actin cytoskeleton (Fig. 5e). Pathogens infection markedly altered the expressive profiles, of which 42 and 142 genes were up- and down-expressed in NV shrimp compared with CK shrimp on 14 dpi (Fig. 5b). For the up-expressed genes in NV shrimp, we clearly observed enrichment in signal and immune pathways. By contrast, metabolic pathways were compromised in NV shrimp on 14 dpi, such as tryptophan and pyruvate metabolisms (Fig. 5f). However, pathogens infection-induced expressions were counteracted by probiotics supplementation, as supported by 10 up-expressed and 27 down-expressed genes in NV shrimp compared with PV shrimp on 14 dpi (Fig. 5c). Impressively, probiotics-induced enrichment of pyruvate metabolism, autophagy, and glycolysis/gluconeogenesis were still detectable in PV shrimp on 14 dpi (Fig. 5g). Nevertheless, we also identified 13 up-expressed and 16 down-expressed genes in PV compared with CK shrimp. Specifically, cGMP-PKG and AMPK signaling pathways were stimulated in PV, whereas cysteine and methionine metabolism, chemokine signaling pathway, and bacterial secretion system exhibited the opposing trend on 14 dpi (Fig. 5d, h). Consistently, probiotics supplementation improved the expression of immune genes (Supplementary Fig. 11a, b), and mitigated pro-inflammatory responses (Supplementary Fig. 11c, d). In particular, the abundances of gut differentially expressed genes (DEGs) were significantly associated with the differentially functional pathways (DFPs) in gut microbiome (Supplementary Fig. 12), indicating a cross-talk between the gut microbiota and shrimp immunity.

### Antagonistic probiotics improve expression of gut tight junction genes

Probiotics supplementation significantly induced (*P* < 0.05, unpaired two-tail *t* test) the expressive level of mucin 6, mucin 2, Rho GTPase-activating protein 17 (ARHGAP17), Ras-related protein Rab-8A (RAB8A) and MAGUK p55 subfamily member 5 (MPP5) (Supplementary Fig. 13). However, pathogens infection significantly compromised the expression of mucin 2, ARHGAP17, RAB8A and MPP5. In contrast, our antagonistic probiotics effectively sustained or even improved the expression of these gut tight junction genes (Supplementary Fig. 13).

### Integrated analysis of pathogens, probiotics, gut microbiome and shrimp transcriptome

**Fig. 6:**
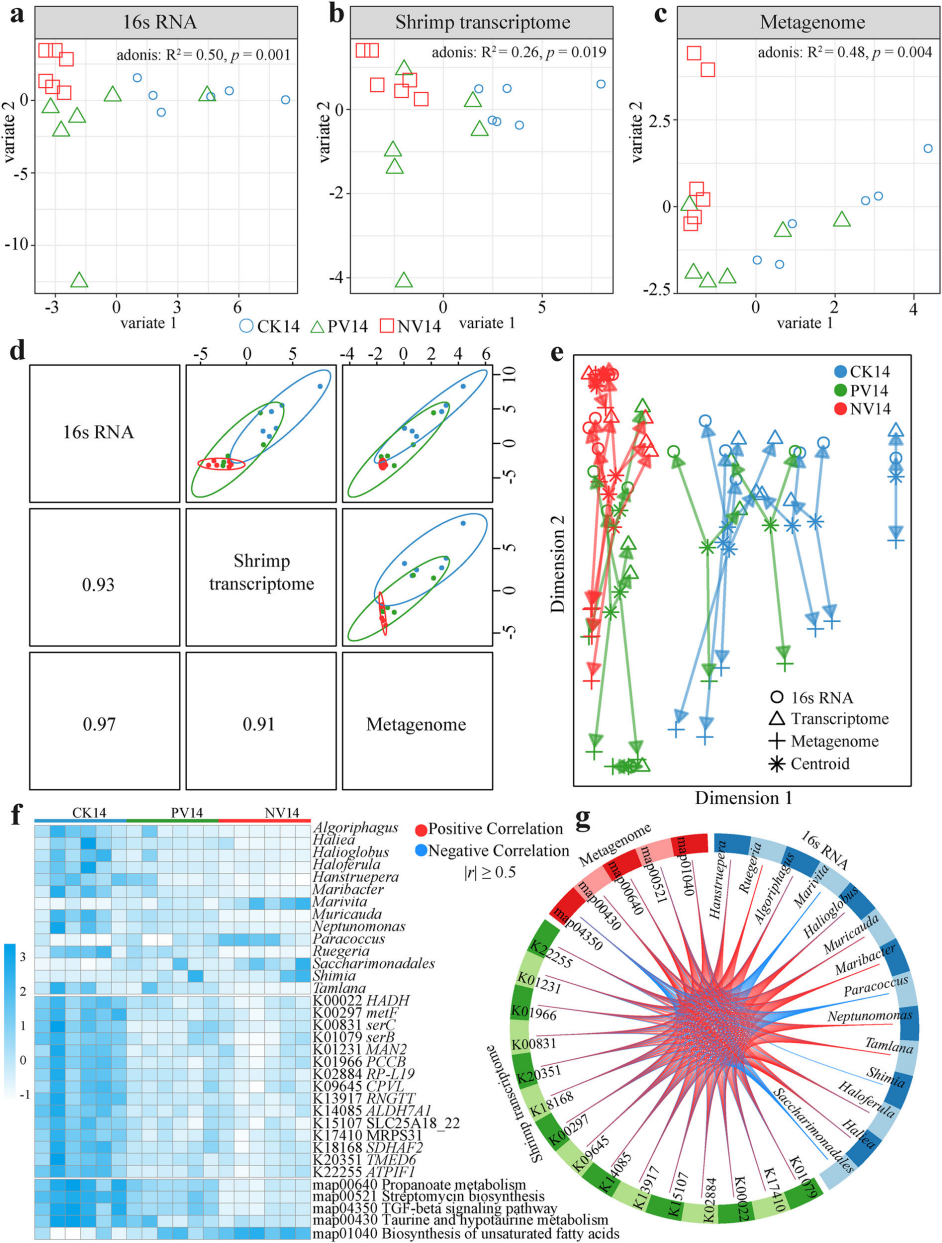
Integrative analysis the three omics data. Sample plots for the **a** compositional, **b** functional structures of gut microbiota, and **c** the gut transcriptome of shrimp. **d** Sample scatterplot displaying the first component in each data set (upper diagonal plot) and Pearson correlation between each component (lower diagonal plot). **e** The arrow plot highlights the agreement between all data sets at the sample level. **f** Clustered image map of the multi-omics signature. Samples are represented in rows, whereas selected features from each of the three omics are represented in columns. **g** The circosPlot showing positive (red lines) and negative (blue lines) correlations (|*r*| ≥ 0.5) between selected features from each dataset (feature names show in each quadrant).

We integrated multiple omics data to identify biologically relevant and robust molecular signatures among the shrimp gut microbiome and transcriptome. Sample plots of DIABLO (Data Integration Analysis for Biomarker discovery using a Latent component method for Omics) model showed that shrimp transcriptome and gut microbiome effectively distinguished the three shrimp cohorts (Fig. 6a−c). The latent components of each omic data were highly correlated (*r* = 0.91 − 0.97) between each other (Fig. 6d), revealing a good agreement among the three data sets at the sample level. The correlation between the components of each data set was maximized as specified in the design matrix. The three kinds of data, representing different levels, exhibited a high correlation at the component level (Fig. 6e). Based on the multi-omics molecular signature, the 18 samples clustered in accordance with the treatment to which the samples belonged (Fig. 6f). The circosPlot visualized the correlation and interactivity of different OMICs data using 14 bacterial genera, 5 KEGG pathways of gut microbiota, and 15 shrimp functional genes, with a correlation coefficient threshold of 0.5, illustrated marked divergences between paired treatments (Fig. 6g).

**Fig. 7:**
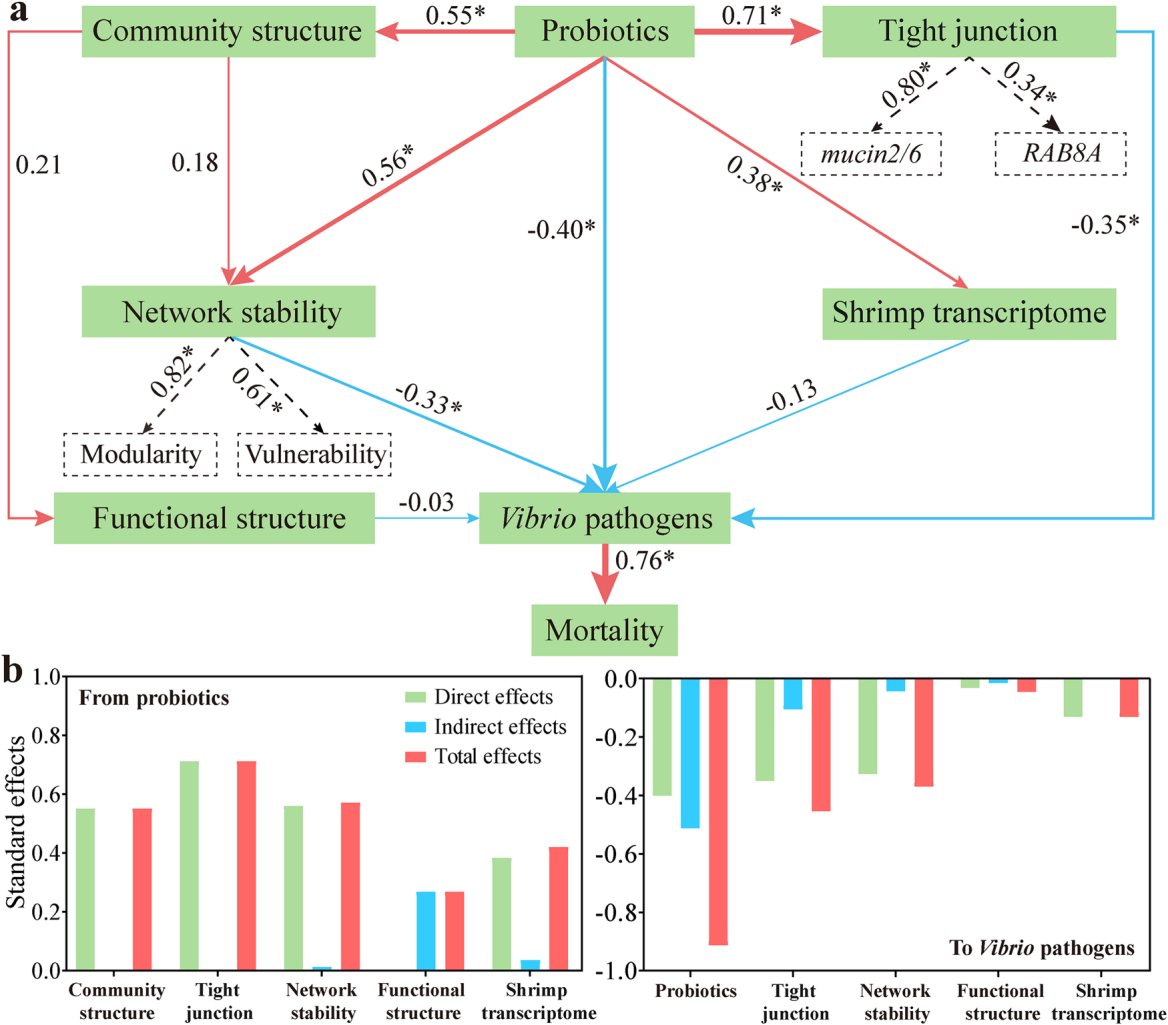
Effects of the driving factors on WFS resistance as determined by a partial least squares path modeling (PLS-PM). **a** PLS-PM showing the cascading relationships of different factors after 1000 bootstraps. The loading for gut network stability and tight junction that create the latent variables are shown in the dashed rectangles. Path coefficients are proportional to the width of the arrow. Red and blue stands indicate positive and negative relationship, respectively. **b** Standardized effects of each factor on summed abundance of pathogens are calculated from PLS-PM. The direct and indirect impacts are summed to form the total effects.

A forward selection procedure identified that antagonistic probiotics, tight junction, shrimp transcriptome, gut microbiome, and gut network stability were the driving factors suppressing the pathogens abundance (Supplementary Table 5). A partial least squares path modeling (PLS-PM) revealed that probiotics supplementation positively governed the gut microbiota (0.55), network stability (0.56), and tight junction (0.71) (Fig. 7a). In addition, probiotics exerted direct and negative suppression (−0.40) on the three *Vibrio* pathogens, as well as indirect effect (−0.51). Meanwhile, network stability (−0.37, direct (−0.33) plus indirect (−0.04) effects) and tight junction (−0.41, direct (−0.31) plus indirect (−0.10) effects) negatively affected the three *Vibrio* pathogens (Fig. 7b). For these reasons, ecological and mechanical barriers jointly suppress pathogen proliferation. Unexpectedly, the overall gut functional structure and shrimp transcriptome negatively affected the pathogens level, although their effects were insignificant (Fig. 7a). Of note, *Vibrio* pathogens strongly and positively (0.76) caused shrimp mortality (Fig. 7a). Overall, probiotics supplementation protects shrimp from WFS through directly suppressing pathogens and indirectly enhancing gut network stability and tight junction.

## Discussion

Shrimp WFS is a disastrous disease with unknown causal agents^2,7^, and accordingly an efficient strategy is unavailable. Our recent work infers that *V. fluvialis*, *V. coralliilyticus* and *V. tubiashii* are the potential candidates of WFS based on their features of primary colonizers, keystone taxa, and high accuracy in diagnosing WFS^4^. Here, we further verified the causality of co-infection with the three pathogens to WFS according to Koch’s postulates (Fig. 1). One might argue that Vt was unimportant in shrimp WFS owing to its unchanged abundance among the test groups (Fig. 1d). However, a keystone taxa is featured by its intensive interactions with other microbes, rather than sheer abundance^11,27^. Indeed, Vf and Vc inefficiently caused WFS (Supplementary Fig. 1). Furthermore, the three *Vibrio* strains synergetically interacted with each other during infection (Supplementary Fig. 7e). Based on these phenotypic and ecological evidences, the three *Vibrio* species were implicated in shrimp WFS etiology. Currently, probiotics are derived from non-aquatic hosts that are poorly tolerant to shrimp biophysics. To circumvent this obstacle, we screened gut symbionts that exerted the maximal potential toward a healthy gut microbiota. The members and ratios of probiotics strains were designed rationally using an ecological framework (Fig. 2). Impressively, probiotics supplementation efficiently prevented shrimp WFS and mortality (Fig. 1c, d). In accordance, antagonistic probiotics directly suppressed the proliferation of pathogens (Figs. 1e, 7 and Supplementary Fig. 2), and stimulated shrimp immunity and tight junction for barrier against the invading pathogens (Fig. 5, Supplementary Tables 3 and 13). Of note, PV shrimp exhibited higher survival rate, body length and body weight than CK shrimp, although they were also immersed by pathogens (Fig. 1). Thus, our designed probiotics not only guard against WFS, and also improve shrimp yield.

Gut microbiota contributes fundamental roles in barrier against pathogens colonization and subsequent disease outcome^8,28^. Thus, dysbiosis in the gut microbiota is commonly detected in diseased shrimp, as observed here (Supplementary Fig. 4a) and elsewhere^29,30^. One might argue that the gut microbiotas were also differed between PV and CK cohorts. However, PV shrimp harbored less pathogens, higher survival rate and yield than CK individuals (Fig. 1). A microbial community possess the inherent potential to adapt to new environmental conditions by adjusting their structure^31^, thus the altered gut microbiota in PV shrimp could be an alternative healthy status. In accordance, pathogens infection significantly increased the turnover rate and AVD of gut microbiota in NV shrimp. However, these detrimental effects were counteracted by probiotics supplementation (Supplementary Fig. 4c, d). A declined turnover rate of a microbial community could occur when extinction rate of extant taxa is reduced. Conversely, the rapid elimination and substitution of gut commensals are parallel with the decreased stability and reassembly of gut microbiota in response to infection. As a consequence, there were significantly higher inter-individual differences (higher AVD) among NV shrimp compared with CK and PV cohorts (Supplementary Fig. 4d). This pattern is in concordant with the Anna Karenina principle, predicting that healthy cohorts are similar, whereas each diseased individuals is sick in its own way^32^. Based on the holobionts theory, hosts recruit phylogeny-specific gut commensals to improve their fitness^33^. Correspondingly, CK and PV shrimp sourced less gut commensals from corresponding bacterioplankton community, as supported by the decreased fit to neutral model and migration rate compared with NV shrimp (Supplementary Fig. 6a−c), which could be attributed to the attenuated selection of WFS-infected shrimp on external species^34^. Consistent with this assertion, the relative importance of homogenizing selection (e.g., host filtering) in CK shrimp was 33.9%, which markedly decreased to 24.4% in NV shrimp (Supplementary Fig. 6d, f). Meta-analyses have depicted that diverse shrimp diseases induce a consistent increase in stochasticity acting on the gut microbiota^29,35^. Of note, probiotics supplementation substantially potentiated shrimp homogenous selection (60.1%) on external taxa (Supplementary Fig. 6e). Thus, one would predict that lower colonization potential (e.g., lower chance that external taxa successfully colonize into the gut of healthy individuals) could be a driving force underlying the patterns observed. Collectively, our designed probiotics facilitate WFS resistance partially through strengthening the stability of gut microbiota, and shrimp filtering on pathogens.

Gut commensals form a complicated network for suppressing external pathogen invasion and enteric pathogen proliferation, thereby alleviating host disease risk^11,12^. We found that pathogens infection significantly increased network vulnerability, while decreased modularity and robustness, leading to more sensitivity to removal of species in NV shrimp. However, these destructive effects of infection on gut network were mitigated by probiotics supplementation (Fig. 4). Ecologically, a gut microbiota with high robust and stability is less likely to be destructed by infection and is therefore less prone to be knock-on detrimental effects on host health^8^. Consistently, we found that strengthened network stability significantly suppressed the three *Vibrio* pathogens and shrimp mortality (Figs. 1 and 7). Overall, our designed probiotics exert a fresh role in regulating shrimp gut bacterial interactions, which strengthen the interactions among gut commensals and subsequent improve shrimp resistance to WFS.

Pathogens infection significantly suppressed the abundances of genes mapped to tryptophan, propanoate, butanoate, taurine and hypotaurine metabolisms (Fig. 4, Supplementary Figs. 7, 8 and 9). Gut commensals can utilize tryptophan to ameliorate inflammation by promoting colonic goblet cell differentiation and inducing mucin gene expression, thereby sustaining the integrity of gut epithelial barrier^36^. The potential of tryptophan metabolism was inhibited under pathogens infection, while stimulated by probiotics supplementation (Supplementary Fig. 7). Correspondingly, gut integrity was destructed by pathogens infection, but not in PV shrimp (Supplementary Fig. 13). Propanoate and butanoate are key short-chain fatty acids (SCFAs) that are primarily produced by gut bacterial metabolism. Consistently, the genes involved in propanoate and butanoate metabolisms were positively and significantly coupled with the abundances of known SCFAs producers *Butyricicoccus*, *Eubacterium* and *Clostridium* genera (Supplementary Figs. 8 and 9). SCFAs could improve survival rate, immune responses, disease resistance, and gut function in aquatic animals^37^. Moreover, dietary supplementation of SCFAs potentiates shrimp (*L*. *vannamei*) innate immunity and antioxidant capacity, thereby protecting against *V*. *harveyi* infection^38^. Taurine and its metabolites contribute crucial roles in regulating mammalian inflammatory responses through the AMPK-mTOR or TLRs/NF-κB pathway^39^, although the role of endogenous taurine in shrimp is uncertain. However, a recent work reveals that taurine modulates shrimp gut microbiota and immunity, thereby enhancing resistance to *Vibrio* infection^30^. In accordance, we observed strong and positive correlations between the abundances of genes facilitating taurine metabolism and probiotics (Fig. 4b, d).

Gut barrier function includes ecological, mechanical and immunological barriers^40^. Disorganization of the ecological barrier, e.g., dysbiosis in compositional and functional structures (Supplementary Figs. 4 and 7), and destabilized networks (Fig. 3) of the gut microbiota, often causes dysfunction of the immunological and mechanical barriers. Pathogens infection activated shrimp inflammatory responses, with significant enrichment of PI3K-Akt signaling pathway and ECM-receptor interaction, whereas normal immune activities such as lysosome and peroxisome were inhibited (Fig. 5f). Similarly, it has been reported that ECM-receptor interaction is significantly up-regulated in shrimp suffering from hepatopancreatic necrosis disease^41^. In addition, several key metabolism pathways were inhibited in infected shrimp (Fig. 5f), while their by-products are known to contribute essential roles in shrimp gut integrity, anti-oxidative capacity and immunity^36,42^. Again, infection-induced detrimental effects were efficiently mitigated by probiotics supplementation (Fig. 5g), thereby protecting gut barrier function and exerting anti-inflammatory properties in shrimp. Beside, probiotics supplementation significantly activated the regulation of actin cytoskeleton (Fig. 5e). Actin cytoskeleton is a crucial regulator controlling the assembly and function of epithelial adherents and tight junctions^43^. In accordance, the expression level of gut tight junction genes was induced by probiotics supplementation (Supplementary Fig. 13). Taken together, apart from facilitating ecological barrier, our antagonistic probiotics also strengthen shrimp immunological and mechanical barriers, and accordingly, improve shrimp resistance to WFS (Fig. 1).

Antagonistic probiotics directly suppressed the three *Vibrio* pathogens, as well as a strong indirect effect. Meanwhile, antagonistic probiotics potentiated gut network stability and tight junction, which further exerted negative and direct associations with pathogens (Fig. 7). It is known that stabilized interactions among gut commensals improve resistance against infection^44^, whereas less complex and connected networks are associated with diverse shrimp diseases^29^. Here, gut network modularity, robustness and invulnerability were strengthened in PV shrimp (Fig. 3). Additionally, pathogens infection induced gut permeability was counteracted by probiotics supplementation (Supplementary Fig. 13). Increased expression of junction proteins has been reported to strengthen gut barrier function, which further prevents or reverses pathogen effects^44,45^. Thus, it is convincible to infer that our designed probiotics could not directly suppress pathogens proliferation but indirectly protect shrimp against infection by enhancing the gut network stability and tight junction.

**Fig. 8:**
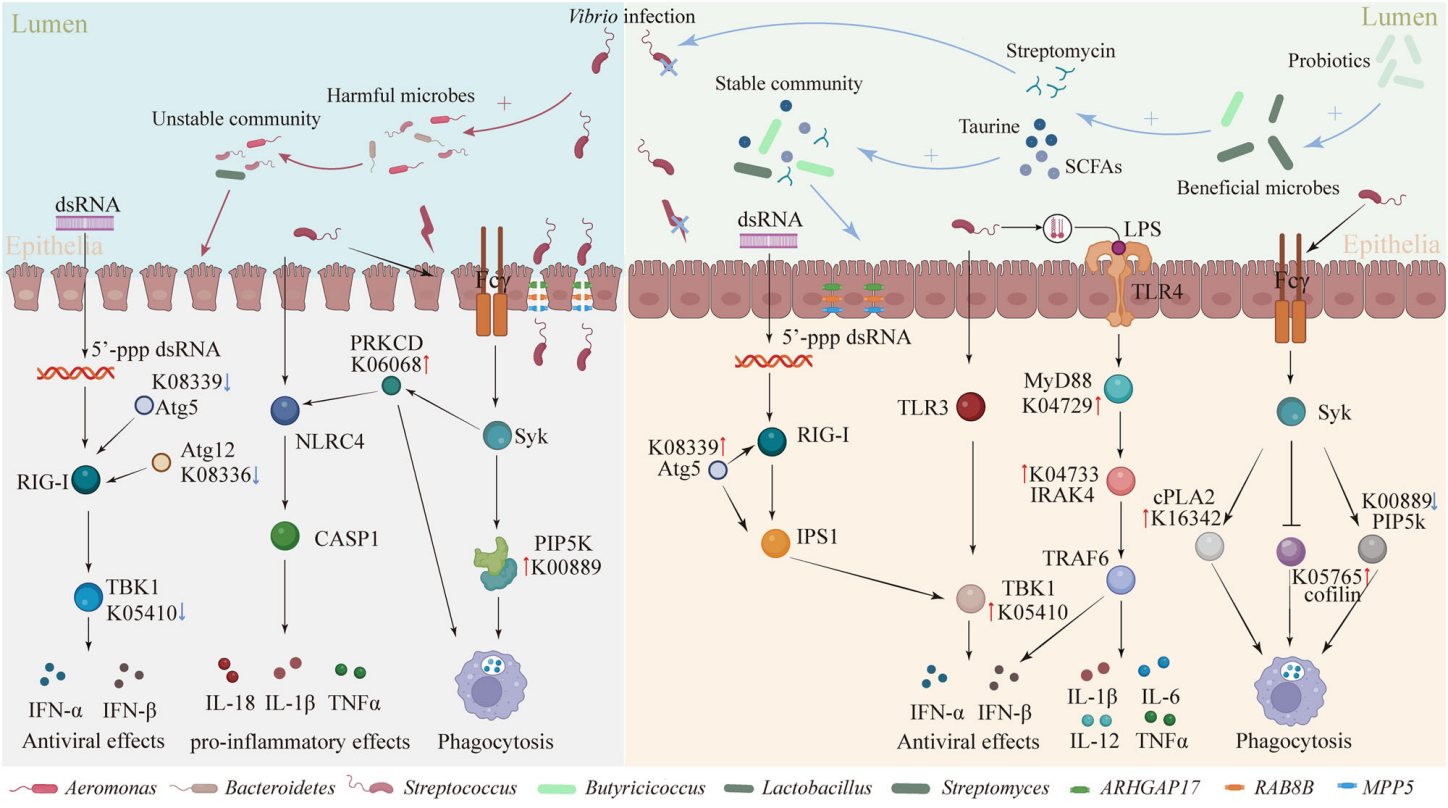
Proposed mechanisms of probiotics protection of shrimp against WFS via the probiotics–gut microbiome–shrimp immunity axis. Probiotics supplementation enriches beneficial gut symbionts (e.g., *Streptomycete*, *Butyricicoccus* and *Clostridium*) that produce sufficient amount of taurine and short-chain fatty acids (SCFAs). Taurine and SCFAs are utilized by gut symbionts to form a more stable network while inducing the expression of gut tight junction proteins (mucin 2 and mucin 6) and stimulating shrimp immune activities, which synergistically prevent, antagonize and kill (e.g., streptomycin) the three *Vibrio* pathogens.

Moving beyond “one pathogen, one disease”, we validate the causal role of co-infection with multiple pathogens in shrimp WFS etiology, and accordingly rationally design antagonistic probiotics preventing WFS. We propose the crosstalk mechanisms that contribute to WFS resistance (Fig. 8). Alterations in the shrimp gut microbiome and transcriptome induced by antagonistic probiotics could control pathogens and prevent shrimp mortality. Probiotics supplementation increases beneficial populations such as *Streptomycete*, *Butyricicoccus* and *Clostridium* genera producing streptomycin, SCFAs and taurine. These by-products could directly kill pathogens and regulate shrimp immune responses. Besides, probiotics supplementation strengthens gut network stability and tight junction, and shrimp selection on external taxa, thereby facilitating ecological and mechanical barriers against pathogens. Moreover, shrimp immune pathways are activated by probiotics supplementation, such as TLRs signaling pathway and Fcγ R-mediated phagocytosis conferring immune barrier (Fig. 8). Collectively, we provide updated frameworks for causally identifying co-infection with multiple pathogens and precisely designing antagonistic probiotics. In addition, our findings markedly deepen the understanding of the beneficial mechanisms of probiotics from the probiotics–gut microbiome–host immunity axis.

## Methods

### Experiment 1: Validating the causal pathogens of shrimp WFS

The three *Vibrio* pathogens of WFS that identified in our previous work^4^ were selectively isolated from the gut of WFS shrimp on the thiosulfate-citrate-bile salts-sucrose agar. The nearly full-length 16S rRNA gene of each strain was amplified using the universal primers 27F and 1492R^46^. The taxonomy of each *Vibrio* strain was assigned by using its 16S rRNA sequence via BLAST search (https://www.ncbi.nlm.nih.gov). We selected the strains with 100% similarity with Vf, Vc or Vt for subsequent experiment.

Pure culture of Vf, Vc or Vt was incubated into liquid Luria Bertani with 2% NaCl at 30°C overnight. The strains were harvested by centrifugation at 4000 rpm for 5 min. The pellets were washed by sterilized normal saline solution (0.9%) for three times. The exact cell density was validated by the dilution plate counting method. Based on the proportions Vf, Vc and Vt in the gut of WFS shrimp, a cocktail of the three pathogens was mixed with a ratio of 7:2:1. Juvenile shrimp were bathed into water containing the pathogens with a final density of 1 × 10^8^ CFU/mL for 14 days. The causal role of the three pathogens in shrimp WFS etiology was validated by disease signs, mortality, and colonization into gut by metagenomics sequencing (Fig. 1).

### Experiment 2: Designing antagonistic probiotics to prevent shrimp WFS

The optimal combination of keystone species that directly antagonized Vf, Vc and Vt were screened by using the dynamic intervention simulation based causal interaction networks (Fig. 2), which integrated the IFE algorithm with the maximum CIS^15^. First, the causal interaction network was constructed using package “DoWhy” (https://microsoft.github.io/dowhy/). The importance of each species in the network was quantified using the Hyperlink-Induced Topic Search (HITS) algorithm. The dynamic intervention simulation (DIS) was further used to evaluate the ability of each key species to restore the diseased microbiota to a normal microbial structure. Finally, we used the IFE algorithm to search for the optimal combination of keystone species for intervention according to the CIS. Accordingly, four keystone strains, *Ruegeria lacuscaerulensis* (Rl), *Nioella nitratireducens* (Nn), *Bacillus subtilis* (Bs) and *Streptomyces euryhalinus* (Se), were determined as the antagonistic probiotics using the metagenomic data in our recent work^19^. The culture media for the four keystone strains were inferred using a web-based Known Media Database (https://komodo.modelseed.org/). Similarly, the taxonomy of each probiotic strain was assigned by as described for the three pathogens. Again, we designed a probiotic cocktail based on their ratio (4:3:2:1) in healthy shrimp gut.

### Experiment 3: Antagonistic probiotics enhance shrimp resistance to WFS

Shrimp were supplemented with our designed probiotic cocktail for 14 days, which were subsequently immersed with the pathogenic cocktail (Fig. 1a). Specifically, 900 healthy post-larvae were randomly dispersed into 18 tanks (50 L), which were fed at 3% of body weight twice a day (at 10:00 and 16:00). The uneaten feed and faeces were removed daily (at 18:00). After one week of acclimatization, the tanks were randomly divided into three treatments, namely, control shrimp (CK), shrimp supplemented with probiotics (10^7^ CFU/g diet for 14 days) plus subsequently immersed with the pathogenic cocktail (PV), and shrimp only immersed with the pathogenic cocktail (NV) (Fig. 1a). Shrimp in PV tanks were fed with probiotics-supplemented diet (10^7^ CFU/g diet) for 14 days. Then, PV and NV groups were bath infected with the pathogenic cocktail. After pathogens immersion, the 18 tanks were monitored every 12 h for 14 days. We collected shrimp samples on 0, 1, 4, 8 and 14 dpi. To assess the effect of rearing water bacterioplankton community on the shrimp gut microbiota, rearing water on 0 dpi (before infection) and 14 dpi were included in our analysis (Fig. 1a, Supplementary Table 2). Approximate 300 ml water for each sample was filtered onto a 0.22 μm membrane (Millipore, Boston, MA, USA) for microbial biomass collection. Gut and hepatopancreas were dissected from each shrimp using sterile forceps at an aseptic workbench. The shrimp used in this study are complied with the Animal Care and Ethics Committee Policies and Guidelines of Ningbo University.

### Analysis of immune and digestive activities

Hepatopancreas tissue was homogenized with four volumes (weight/volume) of ice-cold distilled water and centrifuged at 4 °C with 5000 rpm for 10 min. The supernatant was harvested for measurement of immune (Alkaline phosphatase, lysozyme and peroxidase) and digestive (Pepsin and lipase) activities using corresponding kits (Jiancheng Bioengineering Institute, Nanjing, China), following the manufacturers’ protocols.

### Bacterial 16S rRNA gene amplification, sequencing and data processing

DNA was extracted from shrimp guts using the FAST DNA Spin kit (MOBIO, Carlsbad, CA, USA) according to the manufacturer’s protocols. The extracts were quantified using a NanoDrop ND-2000 spectrophotometer (NanoDrop Technologies, Wilmington, USA). The V3‐V4 regions of the bacterial 16S rRNA gene were amplified using primers 341F and 806R in triplicate. The three amplicons for each sample were combined and purified using a PCR fragment purification kit. The same amounts of purified amplicons from every sample were sequenced using a NovaSeq PE250 platform. Amplicons were processed using the QIIME2 pipeline according to official tutorials^47^. DADA2 was employed to remove errors, noise, and chimeras from the sequences and to assign sequences to ASVs with default parameters^48^. The classify-sklearn naive Bayes taxonomy classifier in the feature-classifier plugin was used to classify the representative sequences of each ASV using the Silva 138 release database. ASVs that were assigned to Chloroplast, Archaea, unclassified, as well as singletons, were excluded from the community. To eliminate the deviation induced by unequal sequencing depths, the sequence number of each sample was rarefied to 16,019 sequences (the lowest sequencing depth across the samples) per sample for subsequent analysis.

### Shotgun metagenomic sequencing and analysis

To infer the effects of probiotics and infection on the functional potentials of the gut microbiome, the 18 shrimp gut samples on 14 dpi were selected for metagenomics sequencing (Supplementary Table 2). Metagenomic library was generated by using a NEBNext Ultra DNA library prep kit (Illumina, San Diego, CA, USA). Index codes were added to attribute sequences to each sample. The 18 samples were sequenced on an Illumina NovaSeq 6000 sequencer (2 × 150 bp) at Magigene Biotech (Shenzhen, China), generating 195.2 Gb raw reads.

The quality of raw sequences was evaluated using FastQC (v0.11.6)^49^ and then quality control using Trimmomatic (v0.38)^50^ for tripping non-biological bases in reads, filtering reads <36 bp and average quality score <20. After quality control, we obtained 171.2 Gb and 5.38 billion high-quality clean reads. Bowtie2 (v2.5.1) was used to exclude host contamination^51^. Kraken2 (v0.38) was employed to assign the microbial taxonomy^52^. The high-quality reads were assembled into contigs using MEGAHIT (v1.2.9)^53^. MEGAHIT uses a “meta-sensitive” mode to assemble the sequence (minimum length >200 bp) by the default setting. The assemblies were evaluated using QUAST (v5.2.0)^54^. Open reading frames (ORFs) were predicted using metaProdigal (v2.6.3)^55^. Then, the abundance of each ORF was quantified by Salmon (v0.11.3)^56^. Finally, DIAMOND (v0.9.18) was adopted to blast ORFs against the KEGG database for function annotation^57^. The fragments per kb per million reads (FPKM) was used to quantify the abundances of unigenes^58^.

### Transcriptomic analysis of gut tissues

Total RNA was extracted from the 30 guts on 0 and 14 dpi (Supplementary Table 2) using TRIzol plus RNA purification kit (Invitrogen, Carlsbad, USA). The concentration and integrity number of total RNA were measured using NanoDrop 2000 spectrophotometer (Thermo Fisher Scientific, Wilmington, USA) and Agilent 2100 Bioanalyzer (Agilent Technologies, Santa Clara, USA), respectively. The quality of total RNA for each sample was further ascertained using the 260/280 ratio (≥1.9) and checked on 1% agarose gel electrophoresis. mRNA was enriched from the total RNA using Oligo (dT) beads that base pair (A-T) with the poly-A before being randomly fragmented into 100–400 bp using an ultra-sonicator. Then, mRNA was reversely transcribed into cDNA using a MGIEasy RNA directional library kit. The cDNA fragments were diluted to 200 ng/μl, then sequencing adapters were added. The transcriptome was sequenced by paired-end sequencing on the Illumina NovaSeq 6000 sequencer at Magigene Biotech (Shenzhen, China).

Transcriptomic data were evaluated and filtered with FastQC (v0.11.6)^49^ to eliminate reads < 75 bp and adapters, leading or trailing bases with Phred base quality (BQ) scores <15, and fragments of every four bases with an average BQ score <20. The filtered reads were aligned to the reference genome of *L. vannamei* (ASM378908v1)^1^ using HISAT2 (v2.2.1)^59^.

### Ecological processes governing the gut microbiota

NCM assumes that all species are ecologically and functionally equivalent, thus community dynamics are controlled by stochastic processes but not by the differences in their competitive abilities. Thus, NCM qualitatively evaluated the importance of stochastic processes on the communities assembly, which frame an abundance-frequency model to characterize the microbial dispersal from the bacterioplankton community metacommunity to shrimp gut microbiota^60^. In this model, the migration rate of “*m*” was calculated using non-linear least-squares fitting by the function “nlsLM” in package “minpack.lm”^61^. The model fitness (*R*^2^) is calculated as the ratio of the sum of squares of the difference between the predicted value and the observed value to the sum of squares of the difference between the observed value and its mean value^62^. Furthermore, to quantitatively infer community assembly mechanisms, a phylogenetic-bin-based null model analysis (iCAMP)^63^ was used to quantify the five community assembly processes (homogeneous selection, heterogeneous selection, dispersal limitation, homogenizing dispersal, drift). This approach includes three major steps: phylogenetic binning, conducting a null model analysis within each bin, and integrating the results of different bins to assess the relative importance of each process^61^.

### Construction of network

To quantitatively compare the probiotics and infection effects on the interspecies interactions in shrimp gut microbiota, binary network was plotted based on a Spearman correlation matrix using package ggClusterNet^64^. Specifically, rare ASVs (mean relative abundance <0.01% across the samples) were eliminated from bacterial communities. The reliable networks (|*r*| > 0.8, *p* < 0.05, Spearman’s rank correlation coefficient test) were visualized using the yfiles plug-in module in the Cytoscape (v. 3.9.1)^65^. Robustness, vulnerability and modularity characterize the network stability. Robustness is the ability of a network to maintain its connectivity when a proportion of edges are deleted, which is discriminated by natural connectivity^66^. The vulnerability of each node measures its relative contribution to the global efficiency. Network vulnerability is indicated by the maximal vulnerability of nodes in the network^67^. Modularity estimates the degree to which a network is compartmentalized into different modules, thus the higher modularity indicates the higher stability of network^12^.

### Multiple-omics integrative analysis

A multivariate dimension reduction discriminant analysis, DIABLO was used to identify biologically relevant and highly correlated signatures from various OMICs data using package “mixOmics”^68^. Sample plots display the component scores, and therefore visualize similarities between samples in a reduced dimensional space spanned by the first few latent components of the model. The cimDIABLO function is a clustered image map specifically implemented to represent the multi-omics molecular signature expression for each sample. The circosPlot represents the correlations between variables of different omics, represented on the side quadrants.

### PLS-PM analysis

A forward selection and adjusted *r*^2^ selection criterion (999 permutations) was used to identify the most important variables impacting the pathogens abundance in a distance‐based multivariate linear model (DistLM)^69^. Then, the same subset of variables was implemented in PLS-PM to quantify the interrelationships among different variables on the pathogens abundance and subsequent shrimp mortality using package “plsmp”^70^. The a priori and theoretical assumptions made to establish the PLS-PM were as follows: (a) Probiotics directly suppressed the pathogens abundance, and (b) probiotics altered the gut microbiome, enhanced the network stability and strengthened the gut tight junctions, which improve shrimp WFS. We used the gut microbiome taxonomic, functional potentials, and shrimp gene expression profiles as a proxy for community structure, functional structure and shrimp transcriptome, respectively. In addition, network stability was reflected by modularity and vulnerability. Tight junction was indicated by mucin 2, mucin 6 and RAB8A.

### Statistical analysis

Ecological approaches were employed to explore the importance of probiotics in barrier against infection in R v3.6.3, unless stated otherwise^71^. A flow-chart roughly showed the employed methods and corresponding purposes in Supplementary Figure 14. In short, PERMANOVA was applied to qualify the relative contributions of probiotics, infection and, dpi, as well as their interactions on the variances in gut microbiota using the “adonis” function in package “vegan”^72^. The temporal turnover rate of gut microbiota was estimated using the time-similarity decay relationship^73^, which tested whether communities are undergoing directional change. To improve statistical power, here we employed tanks (the origin of shrimp samples, tanks served as a conditional factor) as replicates, thus enabled us to test significance in turnover rate between groups using a unpaired *t* test^73^. Phenotypes of bacterial communities were inferred by BugBase (https://bugbase.cs.umn.edu/)^74^. To enable phenotypic inference, taxonomic identities of ASVs were assigned against the Greengenes database (gg_13_5) with 97% cutoff. The false discovery rate (FDR) control method was used to ensure the high quality of DEGs of transcripts. A threshold of unigenes with FDR < 0.05 and |log_2_ Fold change| ≥ 1 was used to identify the DEGs^75^. Similarly, DFPs of the gut microbiota were identified with thresholds of FDR < 0.05 and |log_2_ Fold change| ≥ 2.

### Reporting summary

Further information on research design is available in the Nature Research Reporting Summary linked to this article.

### Supplementary information

Supplemental tables and figures

Reporting Summary

## Data Availability

Raw sequence data obtained in this study have been deposited in Genome Sequence Archive in the BIG Data Center, Chinese Academy of Sciences under accession codes CRA012037 at http://bigd.big.ac.cn/gsa. All other data are contained within the main manuscript and supplemental files.
