# Supplementary material for: Rationally designed probiotics prevent shrimp white feces syndrome via the probiotics–gut microbiome–immunity axis
Source: NPJ Biofilms Microbiomes. 2024 Apr 11;10:40. doi: 10.1038/s41522-024-00509-5 (PMC11009345; doi:10.1038/s41522-024-00509-5)

Supplemental data summary

npj Biofilms and Microbiomes

Rationally designed probiotics prevent shrimp white feces syndrome via the probiotics–gut microbiome–immunity axis

Haonan Sha[1, 2], Jiaqi Lu[1, 2], Jiong Chen[1, 2], Jinbo Xiong[1, 2, *]

[1] State Key Laboratory for Managing Biotic and Chemical Threats to the Quality and Safety of Agro-products, Insititute of Plant Virology, Ningbo University, Ningbo 315211, China

[2] School of Marine Sciences, Ningbo University, Ningbo 315211, China

***Corresponding author**

Jinbo Xiong, E-mail address: xiongjinbo@nbu.edu.cn

Supplementary Table 1 List of the top 100 keystone strains in the network between healthy and white feces syndrome (WFS) shrimp. The combination of the four keystone strains (blue colored) contributed the greatest combination intervention score (CIS = 0.807) by using Iterative Feature Elimination. Intervention scores (IS) indicated the performance of the intervention by each candidate species in dynamic intervention simulation.

Amplicon sequence variants (ASVs)	Control_hits_pval	WFS_hits_pval	Control_abun (%)	WFS_abun (%)	Control_hits_rank	IS	CIS
ASV_113: *Ruegeria lacuscaerulensis*	0.002	0.001	0.414	0.095	1	0.416	0.416
ASV_51: *Bacillus subtilis*	0.001	0.005	0.270	0.067	2	0.332	0.659
ASV_120: *Nioella nitratireducens*	0.001	0.001	0.346	0.018	3	0.324	0.763
ASV_50: *Streptomyces euryhalinus*	0.001	0.006	0.124	0.030	4	0.272	0.807
ASV_384	0.014	0.329	0.016	0.004	36	-0.019	0.831
ASV_251	0.808	0.012	0.025	0.016	11	0.141	0.843
ASV_341	0.856	0.932	0.003	0.025	30	0.091	0.847
ASV_277	0.112	0.848	0.023	0.013	65	0.226	0.850
ASV_163	1.000	0.913	0.023	0.057	18	0.042	0.854
ASV_118	0.307	0.992	0.082	0.053	9	0.042	0.857
ASV_75	0.553	0.823	0.043	0.198	86	0.109	0.860
ASV_55	0.915	0.002	0.211	0.128	16	-0.181	0.864
ASV_83	0.692	0.002	0.058	0.150	34	0.201	0.867
ASV_23	0.902	0.999	0.859	0.134	80	0.000	0.870
ASV_400	0.582	0.922	1.892	0.806	25	0.037	0.874
ASV_395	0.752	0.935	0.019	0.001	89	0.125	0.877
ASV_394	0.822	0.655	0.004	0.017	39	0.033	0.880
ASV_399	0.623	0.961	0.087	0.266	57	0.002	0.884
ASV_397	0.855	0.042	0.003	0.006	97	0.212	0.887
ASV_393	0.854	0.999	0.006	0.014	42	0.015	0.890
ASV_392	0.646	0.978	0.012	0.008	20	0.033	0.894
ASV_391	0.087	0.644	0.014	0.006	37	0.187	0.897
ASV_390	0.892	0.995	0.020	0.000	59	0.009	0.900

ASV_387	0.340	0.002	0.006	0.015	55	0.114	0.904
ASV_385	0.931	0.862	0.017	0.003	23	0.031	0.907
ASV_389	0.950	0.865	0.011	0.010	93	0.016	0.910
ASV_383	0.943	0.365	0.011	0.010	49	0.023	0.914
ASV_382	0.944	0.971	0.008	0.013	67	0.033	0.917
ASV_376	0.948	0.258	0.009	0.013	84	0.030	0.920
ASV_374	0.942	0.104	0.021	0.001	33	0.080	0.924
ASV_371	0.407	0.055	0.006	0.017	92	0.211	0.927
ASV_370	0.883	0.973	0.011	0.012	14	0.012	0.930
ASV_373	0.950	0.987	0.007	0.016	41	0.064	0.934
ASV_369	0.862	0.126	0.013	0.009	48	0.106	0.937
ASV_368	0.001	0.386	0.007	0.016	21	0.230	0.940
ASV_361	0.128	0.123	0.001	0.024	27	0.071	0.944
ASV_356	0.959	0.045	0.014	0.010	38	0.152	0.947
ASV_355	1.000	0.869	0.010	0.015	45	-0.005	0.950
ASV_347	0.011	0.608	0.011	0.015	5	0.192	0.953
ASV_360	0.001	0.369	0.009	0.015	40	0.284	0.957
ASV_344	0.121	0.001	0.009	0.018	75	0.277	0.960
ASV_343	0.892	0.285	0.025	0.002	12	0.011	0.963
ASV_346	0.927	0.813	0.016	0.011	87	0.040	0.967
ASV_339	0.368	0.005	0.010	0.018	19	-0.095	0.970
ASV_338	0.952	0.081	0.012	0.015	13	0.261	0.973
ASV_331	0.867	0.002	0.027	0.001	43	0.406	0.977
ASV_324	0.354	0.827	0.017	0.012	88	0.204	0.980
ASV_323	0.005	0.001	0.004	0.026	77	0.166	0.983
ASV_322	0.009	0.314	0.014	0.015	26	0.103	0.986
ASV_321	0.825	0.13	0.013	0.017	44	-0.003	0.990
ASV_342	0.374	0.686	0.020	0.007	53	0.130	0.993
ASV_333	0.054	0.961	0.013	0.015	15	0.169	0.996
ASV_317	0.252	0.125	0.013	0.016	63	0.105	1.000
ASV_315	0.325	0.527	0.013	0.018	51	0.028	1.000
ASV_328	0.058	0.321	0.017	0.011	52	0.191	1.000
ASV_312	0.565	0.537	0.029	0.002	60	0.048	1.000
ASV_326	1.000	0.995	0.011	0.019	69	0.029	1.000

ASV_313	0.112	0.662	0.007	0.024	46	0.136	1.000
ASV_310	0.915	0.011	0.017	0.015	61	0.137	1.000
ASV_236	0.001	0.518	0.045	0.001	22	0.168	1.000
ASV_308	0.006	0.491	0.018	0.013	54	0.159	1.000
ASV_283	0.002	0.396	0.012	0.024	85	0.134	1.000
ASV_307	0.012	0.888	0.009	0.023	74	0.146	1.000
ASV_231	0.104	0.001	0.010	0.038	47	0.170	1.000
ASV_263	0.164	0.597	0.018	0.021	64	0.157	1.000
ASV_238	0.132	0.889	0.010	0.035	81	0.234	1.000
ASV_287	0.203	0.194	0.028	0.007	58	0.268	1.000
ASV_265	0.330	0.368	0.020	0.019	94	0.042	1.000
ASV_279	0.407	0.997	0.035	0.001	62	0.041	1.000
ASV_230	0.365	0.001	0.024	0.024	82	0.211	1.000
ASV_306	0.638	0.893	0.016	0.016	70	0.120	1.000
ASV_260	0.747	0.975	0.017	0.023	50	0.065	1.000
ASV_233	0.765	0.032	0.041	0.005	56	0.086	1.000
ASV_290	0.760	0.001	0.017	0.018	95	0.319	1.000
ASV_300	0.817	0.999	0.009	0.025	31	0.024	1.000
ASV_270	0.800	0.848	0.009	0.030	98	0.184	1.000
ASV_241	0.869	0.352	0.030	0.015	8	0.020	1.000
ASV_255	0.886	0.002	0.015	0.026	35	0.218	1.000
ASV_267	0.875	0.994	0.032	0.006	91	0.070	1.000
ASV_254	0.848	0.855	0.022	0.019	78	0.056	1.000
ASV_286	0.884	0.999	0.016	0.019	90	0.007	1.000
ASV_244	0.861	0.997	0.040	0.003	99	0.032	1.000
ASV_275	0.881	0.982	0.013	0.024	100	0.035	1.000
ASV_269	0.909	0.313	0.001	0.039	6	0.015	1.000
ASV_298	0.896	0.659	0.032	0.001	66	0.131	1.000
ASV_259	0.914	0.842	0.035	0.005	83	0.069	1.000
ASV_247	0.936	0.506	0.017	0.025	28	0.205	1.000
ASV_288	0.940	0.831	0.002	0.034	10	0.080	1.000
ASV_243	0.936	0.001	0.039	0.004	71	-0.210	1.000
ASV_266	1.000	0.632	0.021	0.018	68	0.222	1.000
ASV_284	1.000	0.969	0.035	0.001	72	0.165	1.000

ASV_273	1.000	0.756	0.026	0.011	7	0.145	1.000
ASV_274	0.653	0.997	0.008	0.030	73	0.036	1.000
ASV_245	1.000	0.928	0.031	0.012	29	0.029	1.000
ASV_278	1.000	0.618	0.032	0.004	24	0.014	1.000
ASV_282	1.000	0.999	0.021	0.015	79	0.012	1.000
ASV_237	1.000	0.111	0.035	0.010	32	0.007	1.000
ASV_229	0.001	0.512	0.019	0.029	76	0.000	1.000
ASV_228	0.001	0.483	0.045	0.003	96	0.000	1.000
ASV_227	0.001	0.504	0.026	0.023	17	0.000	1.000

Supplementary Table 2 Study design and the sampling size for each habitat (shrimp gut and rearing water) on a given date. After 7 days of acclimatization, the 18 tanks were randomly divided into 3 treatments: shrimp that did not receive probiotics (negative and healthy controls, CK), shrimp that were supplemented with our designed probiotics and were subsequently immersed with the defined *Vibrio* cocktail (probiotics plus *Vibrio* infection, PV), and shrimp that did not receive probiotics but were challenged with *Vibrio* cocktail (*Vibrio* infection, NV). G represents shrimp gut samples, W represents rearing water samples.

Habitats	Groups	Sampling date					
		26th April	10th	11th	14th	18th	24th May
Gut	GCK	6	6	6	6	6	6
	GPV	/	6	6	6	6	6
	GNV	/	/	6	6	6	6
Water	WCK	/	6	/	/	/	6
	WPV	/	6	/	/	/	6
	WNV	/	/	/	/	/	6
Days along experiment		-14	0	1	4	8	14

Supplementary Table 3 Quantitative the effects of probiotics, pathogens infection and time (days post infection, dpi) on the variations in the shrimp gut bacterial community using permutational multivariate analysis of variance (perMANOVA) with adonis function. The R^2 value is the proportion of the community variances constrained by each variable or interaction.

	F. Model	R^2	*P*
Time (dpi)	6.28	0.063	< 0.001
Probiotics	4.69	0.047	< 0.001
Infection	5.15	0.051	< 0.001
Time : Infection	2.70	0.027	0.008
Time : Probiotics	2.06	0.021	0.039
Infection : Probiotics	2.28	0.023	0.011

Supplementary Table 4 Metadata statistics obtained from metagenomes. The numbers represent biological replicates for each treatment. Refer to Supplementary Table 2 for abbreviations.

Sample	Sequence	Base (GB)	Q20 (%)	Q30 (%)	Percentage (%)
CK1	29163373	9.28	97.5	92.9	69.6
CK2	32375901	10.6	98.5	95.0	91.3
CK3	35598783	11.9	98.7	95.6	95.3
CK4	29204682	9.36	98.4	95.0	83.2
CK5	27197163	8.58	97.0	91.9	59.4
CK6	31962609	10.3	98.4	94.9	88.1
PV1	25457211	8.03	98.1	94.3	69.7
PV2	29342626	9.24	97.6	93.2	72.9
PV3	29563950	9.36	97.0	91.8	53.3
PV4	29125931	9.08	96.6	91.2	57.5
PV5	32000331	10.1	97.6	93.2	76.0
PV6	26007965	7.99	97.0	92.0	54.9
NV1	30205409	9.51	96.7	91.2	59.5
NV2	28721295	9.05	97.1	91.9	45.9
NV3	31096512	9.90	96.6	90.9	49.3
NV4	30891805	9.75	96.7	91.2	34.7
NV5	31124784	9.93	96.7	91.2	34.3
NV6	29142456	9.24	96.5	90.9	52.9
Average	29899044	9.51	97.4	92.7	74.0

Note: Percentage is the proportion of sequences assigned to shrimp

Supplementary Table 5 Distance-based multivariate analysis for a linear model (DistLM) of pathogens abundance against variables based on forward selection with 999 permutations. The percentage of variation explained by each variable added to model was conditional on variables already in the model.

Variables	F	*P*	Cumulative (%)
Forward selection			
Probiotics	11.7	0.001	26.9
Community structure	6.7	0.001	38.2
Network stability	4.6	0.001	45.5
Functional structure	2.5	0.003	50.6
Tight junction	2.4	0.001	53.8
Shrimp transcriptome	1.6	0.004	55.9

Supplementary Figure 1 The phenotypes of shrimp in response to infections. Shrimp were immersed with one *Vibrio* strain, paired *Vibrio* strains or the three *Vibrio* strains of *V. fluvialis* (Vf), *V. coralliilyticus* (Vc) and *V. tubiashii* (Vt) on 14 days post infection, respectively.

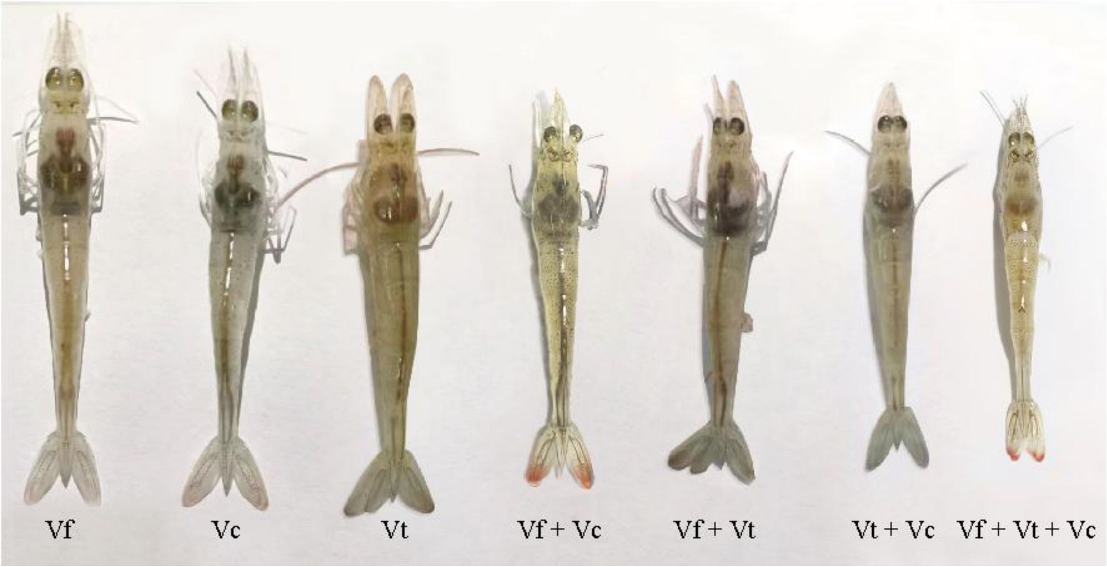

Supplementary Figure 2 Correlations between pathogens and designed probiotics. The negative correlations of the summed abundance of the detected three probiotics (*Ruegeria lacuscaerulensis*, *Nioella nitratireducens*, and *Bacillus subtilis*) and that of three *Vibrio* pathogens (*V. fluvialis*, *V. coralliilyticus*, and *V. tubiashii*) (**a**), the abundance of each *Vibrio* pathogen in CK (**b**), PV (**c**), and NV (**d**) shrimp, respectively. The *p*-values were obtained from linear regression analysis. Refer to Supplementary Table 2 for abbreviations.

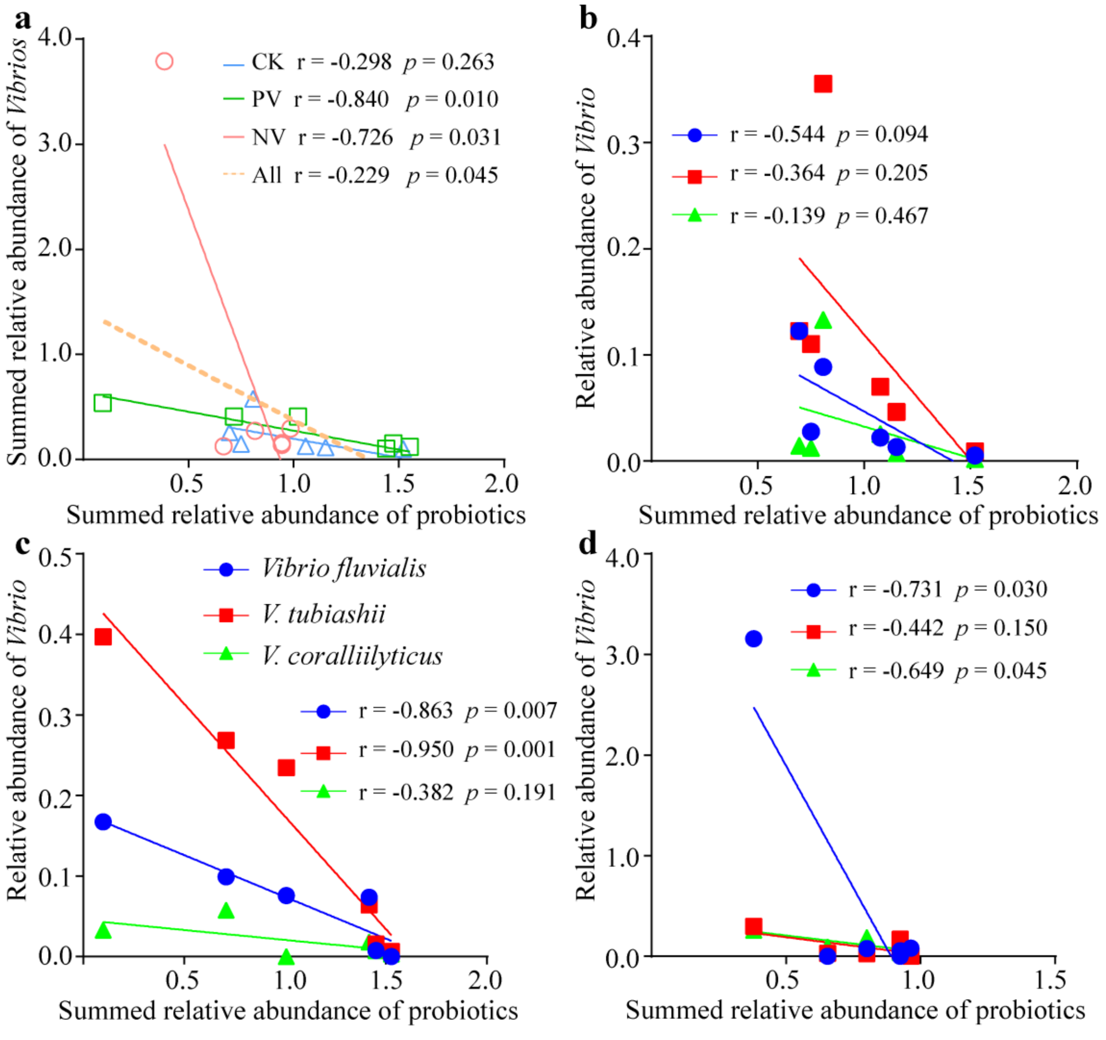

Supplementary Figure 3 Comparison of digestive and immune activities between treatments. **a** Pepsin, **b** Lipase, **c** Peroxidase, **d** Alkaline phosphatase, **c** Lysozyme. Significance in mean ± standard deviation between CK and PV on 0 dpi was tested using unpaired *t* test. Different lowercase letters indicate significant differences among treatments (CK, PV and NV) on 14 dpi using one-way analysis of variance (ANOVA). The numbers represent dpi. Refer to Supplementary Table 2 for abbreviations.

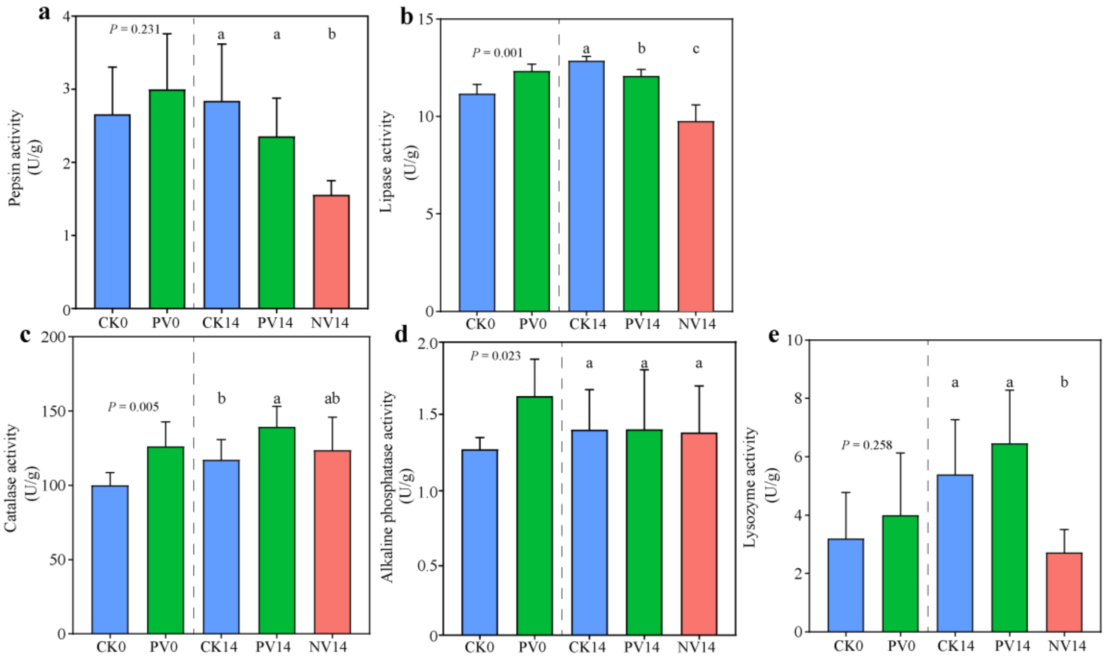

Supplementary Figure 4 Antagonistic probiotics and infection effects on the dynamics of bacterial community. Constrained analysis of principal coordinates depicting the effects of treatments and dpi on the bacterial communities in shrimp gut (**a**) and rearing water (**b**). Time–similarity decay relationships (**c**), average variation degree (**d**), BugBase inferred abundances of anaerobic (**e**), forms biofilms (**f**) and putative pathogens (**g**) of the gut microbiota among treatments. The temporal turnover rate (the regression slope) is estimated using a linear regression fit between the pairwise similarities along dpi. Significance between CK and PV on 0 dpi was test using unpaired *t* test. Different lowercase letters indicate significant differences among treatments on 14 dpi using one-way ANOVA. Refer to Supplementary Table 2 for abbreviations.

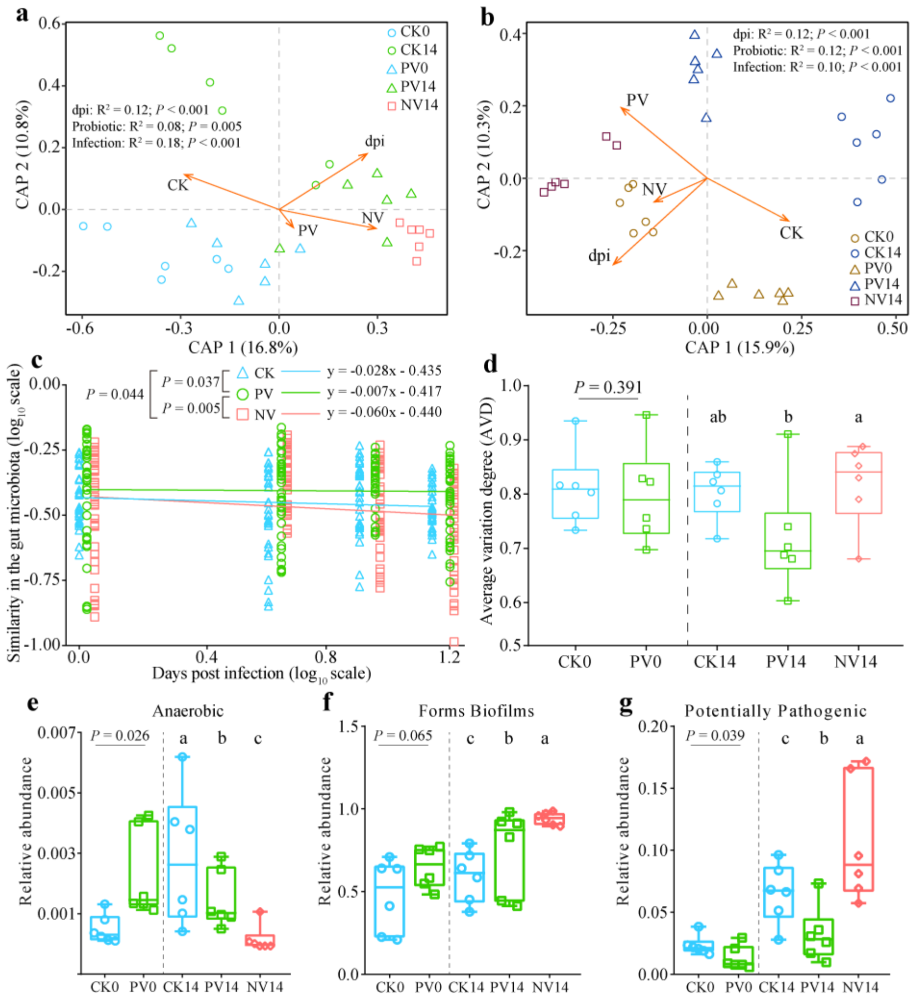

Supplementary Figure 5 Heatmap showing the differentially gut bacterial genera. **a** Enriched 64 genera, **b** depleted 60 genera in CK compared with NV on 14 dpi. The columns on the right are the averaged abundance within each treatment. Refer to Supplementary Table 2 for abbreviations.

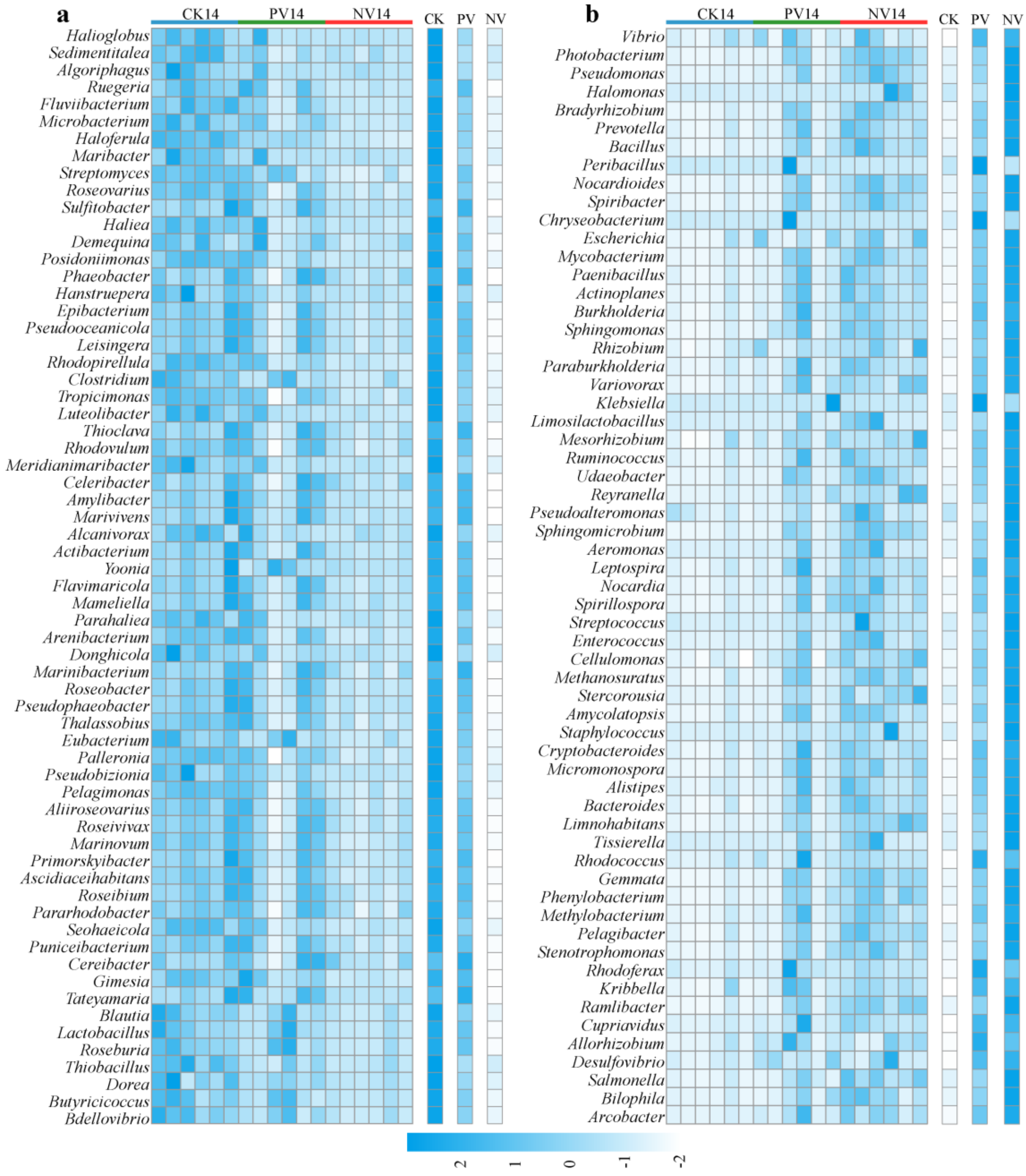

Supplementary Figure 6 Ecological processes governing the gut microbiota. Neutral model applied to the gut microbiota of CK (**a**), PV (**b**) and NV (**c**) shrimp with matched rearing water microbes as the source. The solid line is the best fit to the neutral model, and the dashed lines indicate 95% confidence intervals around the neutral model prediction. R^2 represents the fit to this model, while the *m* value indicates community immigration rate. ASVs that occur more, neutral or less frequently than predicted by the model are shown in blue, green and red, respectively. The contribution of the five ecological processes governing the gut microbiota of CK (**d**), PV (**e**), and NV (**f**) shrimp. Refer to Supplementary Table 2 for abbreviations.

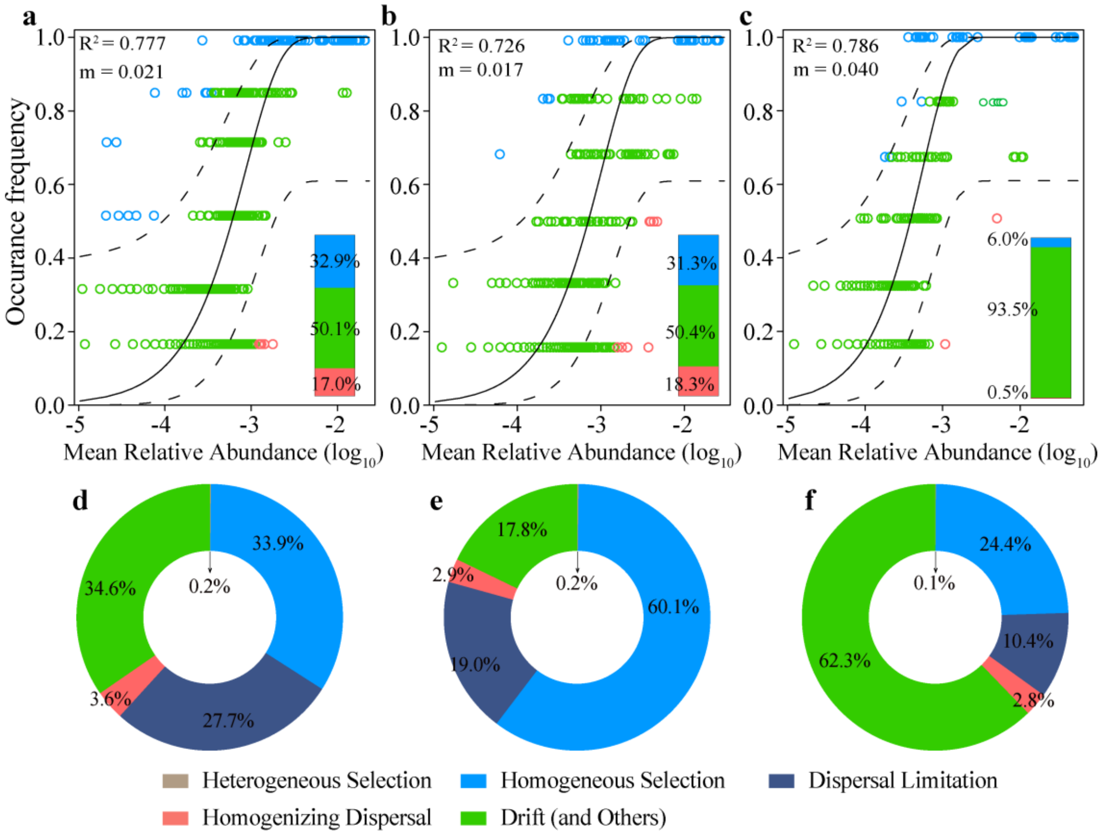

Supplementary Figure 7 Metagenomic analysis of the shrimp gut microbiota. Volcano plot depicting the differentially functional pathways (DFPs) between PV vs. CK (**a**), NV vs. PV (**b**), PV vs. CK (**c**) shrimp on 14 dpi. Numbers indicate the enriched and depleted DFPs. Heatmap (**d**) and co-occurrence network (**e**) depict the correlations among DFPs, (summed) abundance(s) of the three *Vibrio* pathogens and the three detected antagonistic probiotics. Refer to Supplementary Table 2 for abbreviations. The *p*-value was calculated using two-tailed paired *t*-test.

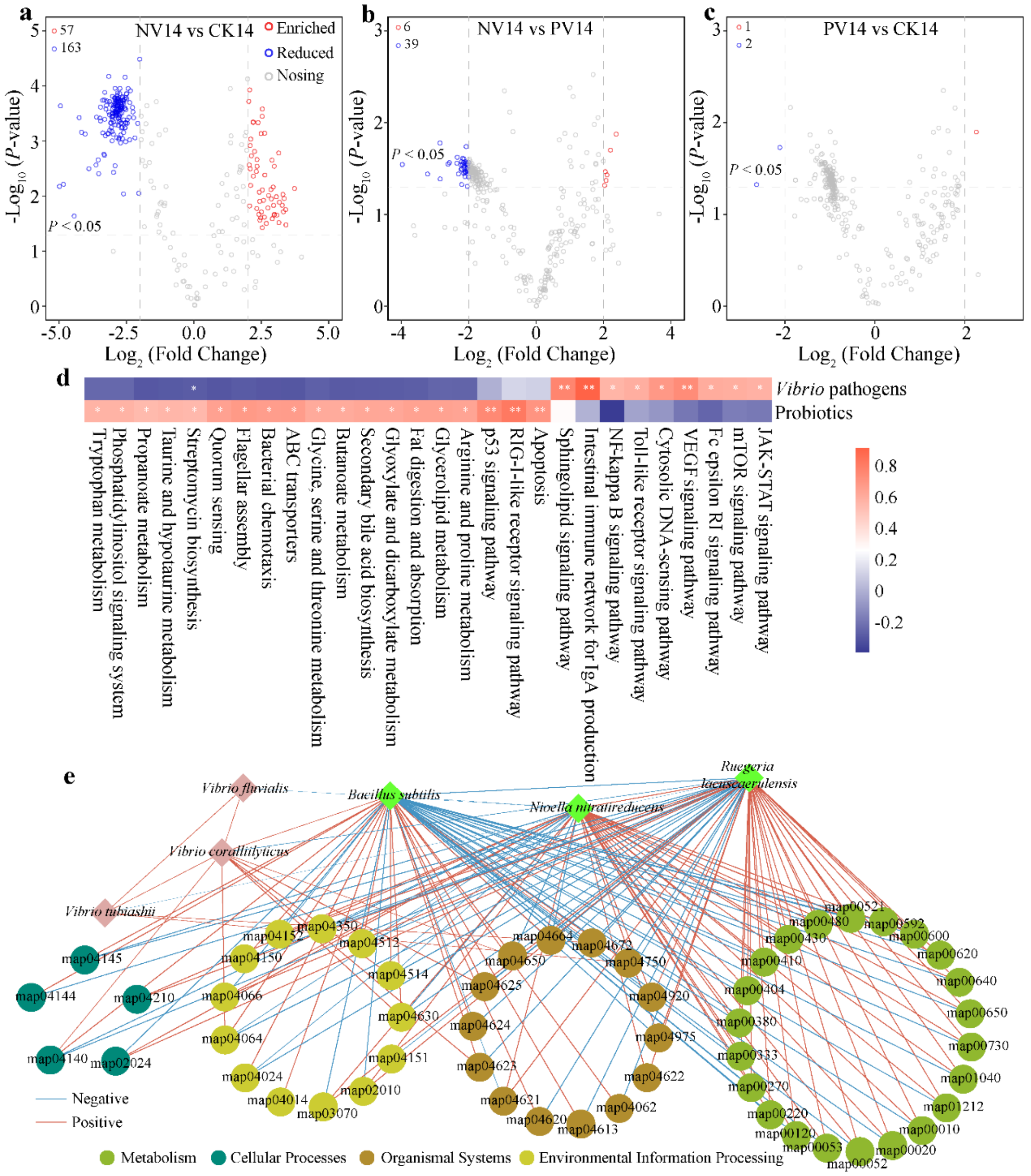

Supplementary Figure 8 Constructed pathway involved in propanoate metabolism. a Mapped pathway of gut microbial propanoate metabolism in shrimp. The downward arrows indicate the differentially functional genes that are depressed in the NV shrimp compared with CK shrimp on 14 dpi. **b** The gut bacterial genera, summed pathogens, or summed probiotics exhibit strong associations with the genes involving in propanoate metabolism. Only the Spearman rank correlations with $P < 0.05$ are considered as strong associations and are marked with "*" in the heatmap. Significance of the relationship was examined by Spearman's *r* correlation.

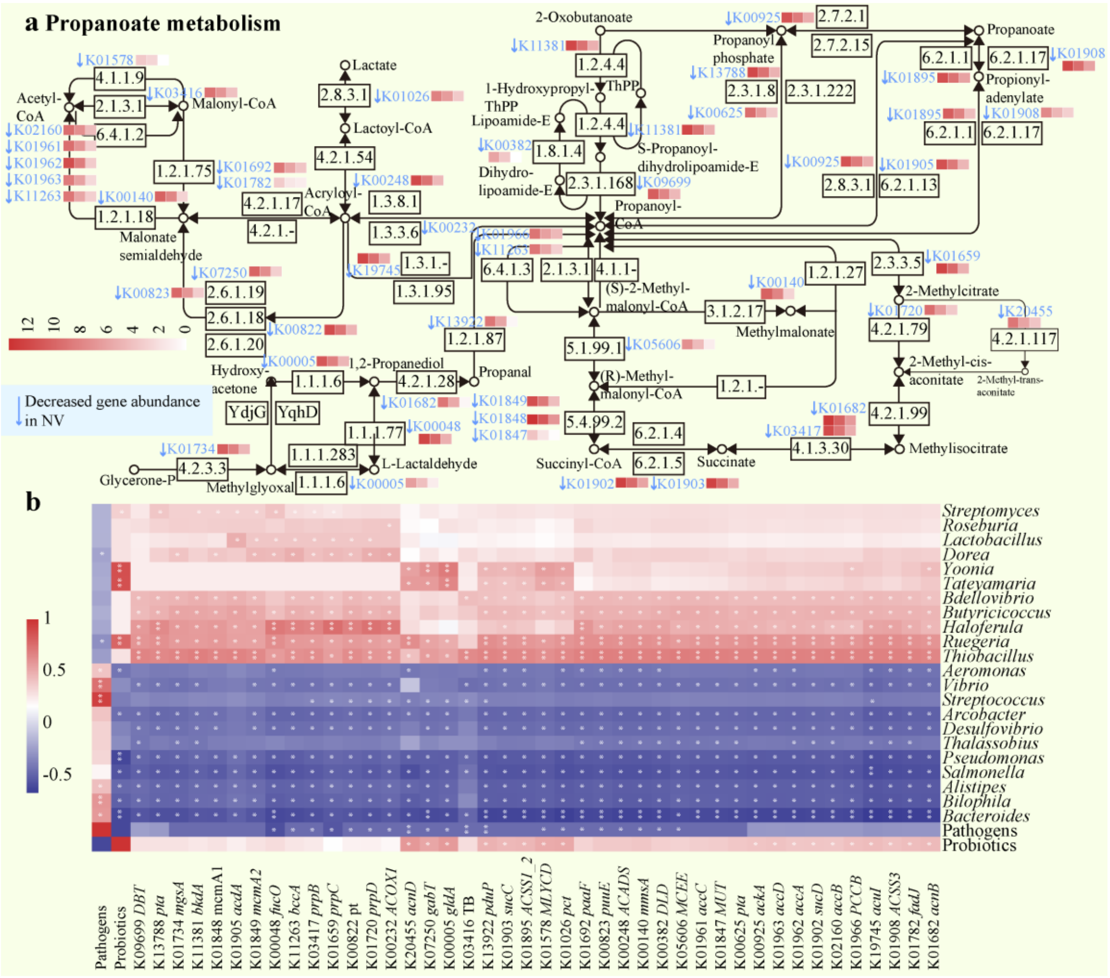

Supplementary Figure 9 Constructed pathway involved in butanoate metabolism. **a** Mapped pathways of butanoate metabolism in shrimp. The downward and upward arrows indicate the differentially functional genes are depressed or enriched in the NV shrimp compared with CK shrimp on 14 dpi, respectively. **b** The gut bacterial genera, summed pathogens, or summed probiotics exhibit strong associations with the genes involving in butanoate metabolism. Only the Spearman correlations with $P < 0.05$ are considered as strong associations and are marked with “*” in the heatmap. Significance of the relationship was examined by Spearman’s r correlation.

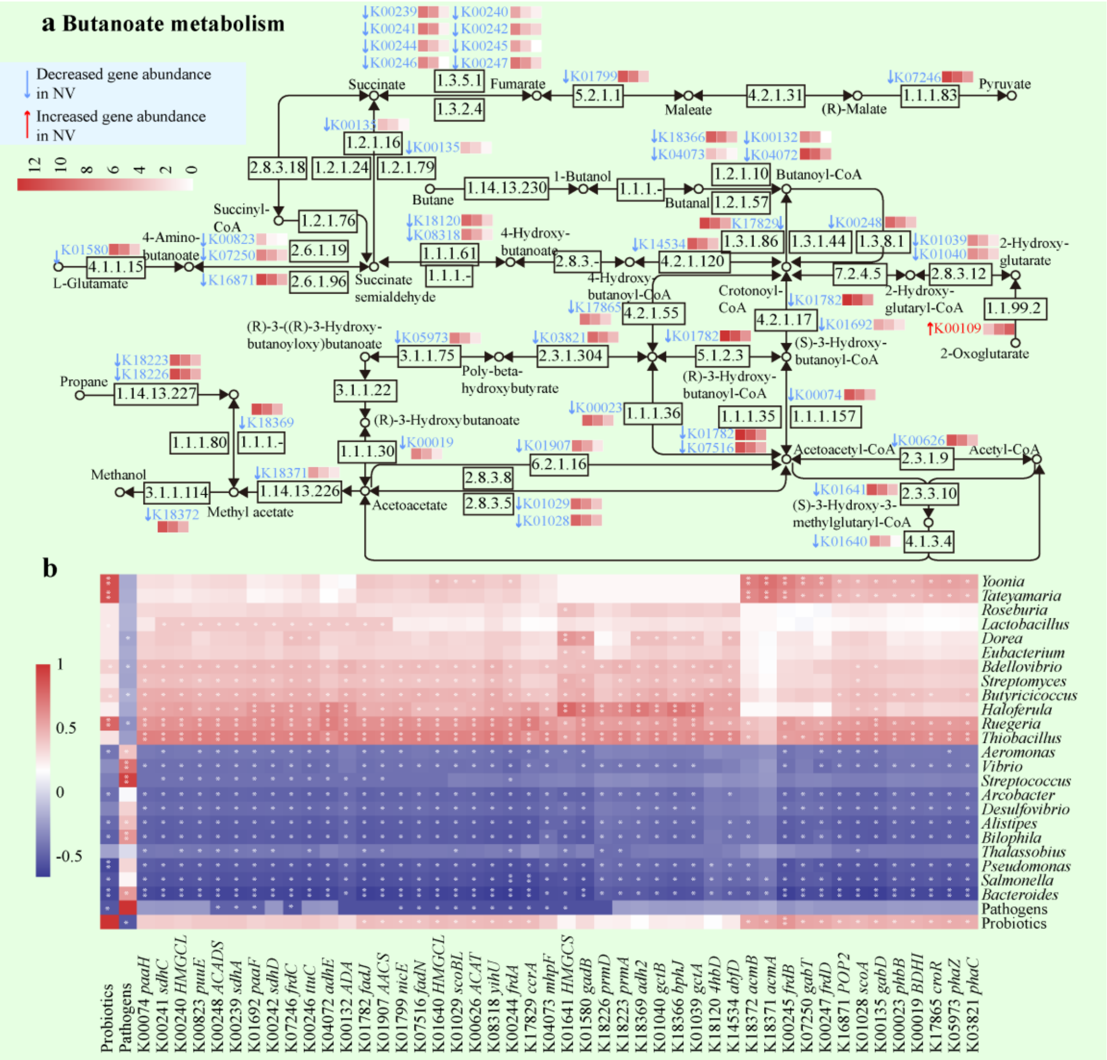

Supplementary Figure 10 Correlations between enzyme activity and matched coding genes. Positive and significant correlation between the activity of lipase (**a**), lysozyme (**b**) or alkaline phosphatase (**c**) and the expressive level of its coding genes, respectively. The *p*-values were obtained from linear regression analysis.

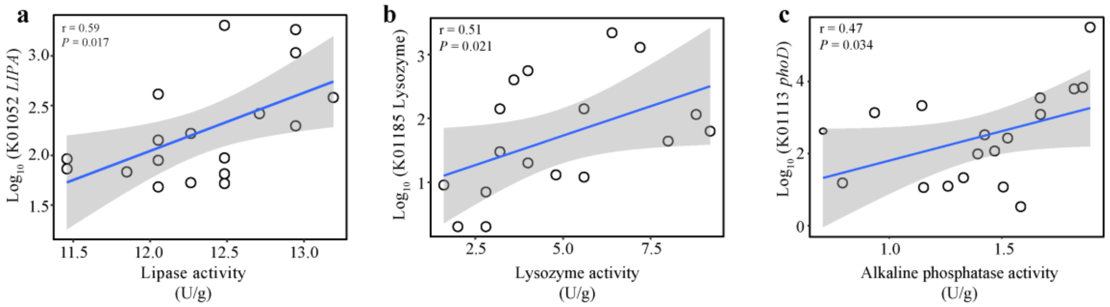

Supplementary Figure 11 Gene ontology (GO) enrichment analysis of genes involving in immunity using transcriptome data. Comparison the expressive levels of (**a**) Toll-like receptor 4 (*TLR4*), (**b**) Myeloid differentiation primary response protein MyD88 (*MyD88*), (**c**) Interleukin 1 beta (*IL-1β*), (**d**) LRR and PYD domains-containing protein 3 (*NLRP3*). Significance between CK and PV on 0 dpi was tested using unpaired *t* test. Different lowercase letters indicate significant differences among CK, PV and NV cohorts on 14 dpi using one-way ANOVA. Refer to Supplementary Table 2 for abbreviations.

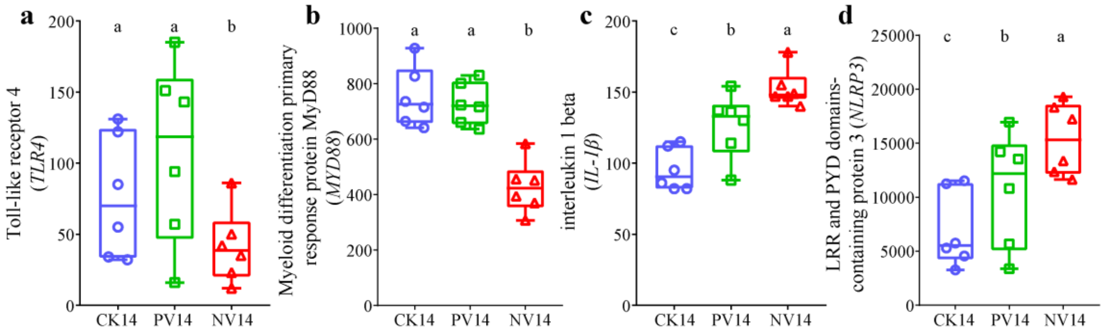

Supplementary Figure 12 The gut differentially expressed genes are strongly associated with the differentially functional pathways in the shrimp gut microbiota. Only the Spearman correlations with $P < 0.05$ are considered as strong associations and are marked with “*” in the heatmap. The red and blue cells in the heatmap indicate positive and negative correlations, respectively. The four mapped pathways are red colored. Significance of the relationship was examined by Spearman’s r correlation.

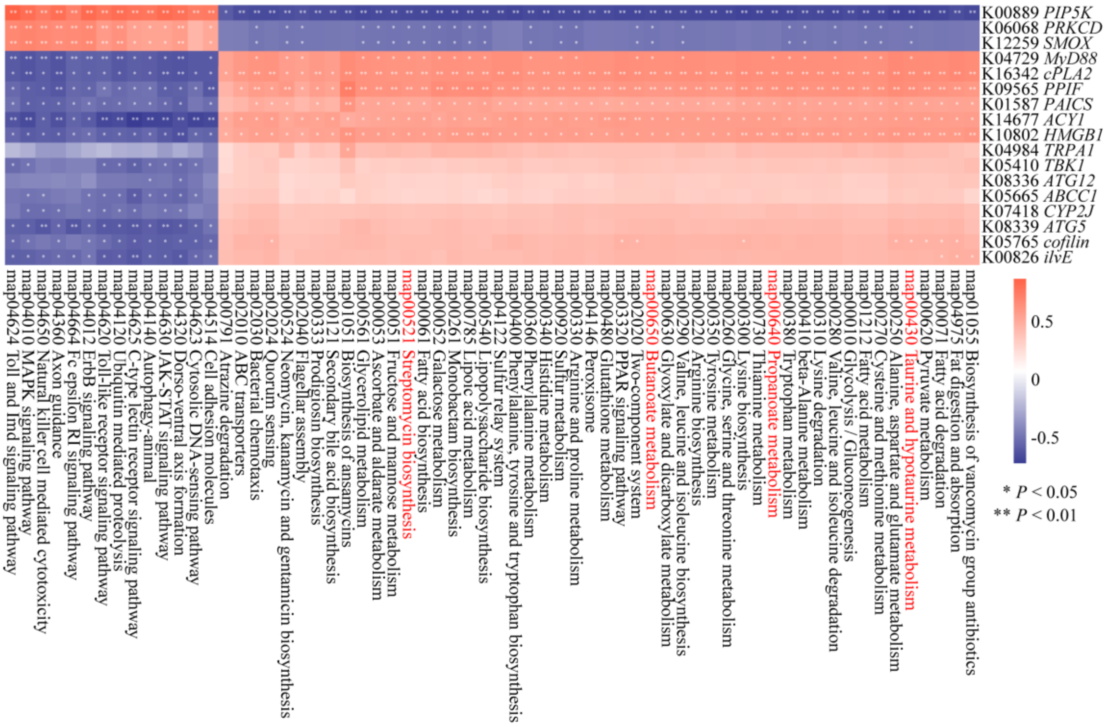

Supplementary Figure 13 Gene ontology (GO) enrichment analysis of genes involving in gut tight junction using transcriptome data. Comparison the expressive levels of gut (**a**) Transforming growth factor beta gene (*TGFBN*), (**b**) mucin 6, (**c**) mucin 2, (**d**) Rho GTPase-activating protein 17 (*ARHGAP17*), (**e**) Ras-related protein Rab-8B (*RAB8B*), and (**f**) MAGUK p55 subfamily member 5 (*MPP5*) among treatments. Significance between CK and PV on 0 dpi was tested using unpaired *t* test. Different lowercase letters indicate significant differences among CK, PV and NV cohorts on 14 dpi using one-way ANOVA. Refer to Supplementary Table 2 for abbreviations.

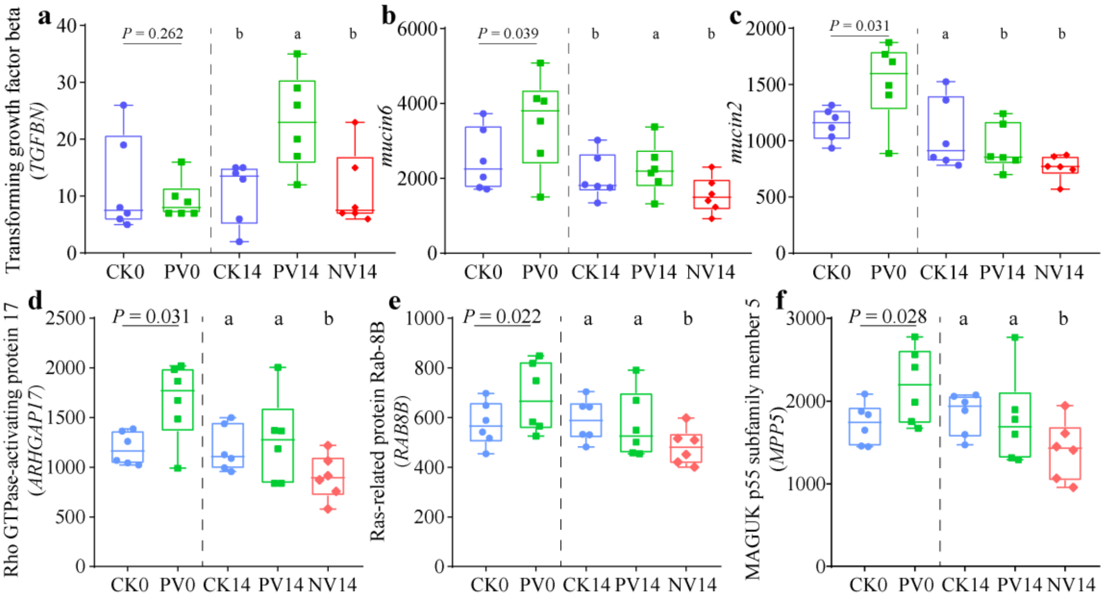

Supplementary Figure 14 A flow-chart roughly shows the employed methods and corresponding purposes.

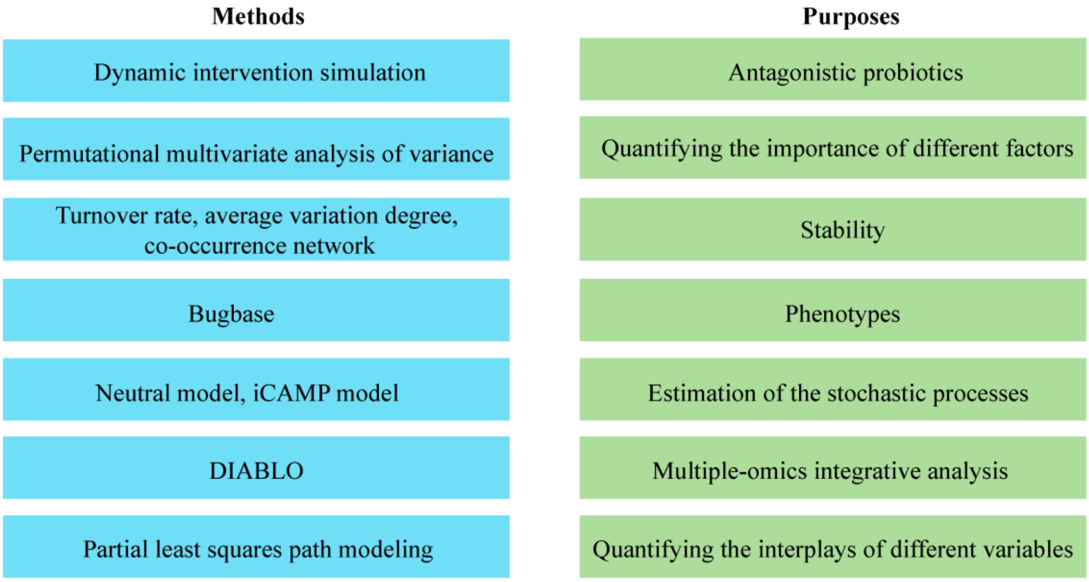

Supplement: Supplementary file 1 — Supplemental tables and figures [file 41522_2024_509_MOESM1_ESM.pdf]
